# Novel Self‐Assembled Multifunctional Nanoprobes for Second‐Near‐Infrared‐Fluorescence‐Image‐Guided Breast Cancer Surgery and Enhanced Radiotherapy Efficacy

**DOI:** 10.1002/advs.202205294

**Published:** 2023-01-31

**Authors:** Yong‐Qu Zhang, Wan‐Ling Liu, Xiang‐Jie Luo, Jun‐Peng Shi, Yun‐Zhu Zeng, Wei‐Ling Chen, Wen‐He Huang, Yuan‐Yuan Zhu, Wen‐Liang Gao, Rong‐Hui Li, Zi‐He Ming, Li‐Xin Zhang, Rui‐Qin Yang, Jia‐Zheng Wang, Guo‐Jun Zhang

**Affiliations:** ^1^ Department of Breast‐Thyroid‐Surgery and Cancer Center Xiang'an Hospital of Xiamen University School of Medicine Xiamen University No. 2000 East Xiang'an Road Xiamen 361100 China; ^2^ Fujian Key Laboratory of Precision Diagnosis and Treatment in Breast Cancer (Xiang'an Hospital of Xiamen University) No. 2000 East Xiang'an Road Xiamen 361100 China; ^3^ Xiamen Key Laboratory of Endocrine‐Related Cancer Precision Medicine No. 2000 East Xiang'an Road Xiamen 361100 China; ^4^ College of Chemistry and Chemical Engineering Xiamen University No. 422 South Siming Road Xiamen 361005 China; ^5^ Xiamen Institute of Rare Earth Materials Haixi Institute Chinese Academy of Sciences No. 255 South DuiYing Road Xiamen 361001 China; ^6^ Department of Pathology Cancer Hospital of Shantou University Medical College No. 7 Raoping Road Shantou 515000 China; ^7^ Xiamen Research Center of Clinical Medicine in Breast & Thyroid Cancers No. 2000 East Xiang'an Road Xiamen 361100 China; ^8^ Department of Medical Oncology Xiang'an Hospital of Xiamen University No. 2000 East Xiang'an Road Xiamen 361100 China; ^9^ Cancer Research Center School of Medicine Xiamen University No. 4221 South Xiang'an Road Xiamen 361100 China

**Keywords:** breast cancer, multifunctional nanoprobes, radiosensitization, second near‐infrared‐fluorescence imaging, surgical navigation

## Abstract

Breast‐conserving surgery (BCS) is the predominant treatment approach for initial breast cancer. However, due to a lack of effective methods evaluating BCS margins, local recurrence caused by positive margins remains an issue. Accordingly, radiation therapy (RT) is a common modality in patients with advanced breast cancer. However, while RT also protects normal tissue and enhances tumor bed doses to improve therapeutic effects, current radiosensitizers cannot meet these urgent clinical needs. To address this, a novel self‐assembled multifunctional nanoprobe (NP) gadolinium (Gd)–diethylenetriaminepentaacetic acid–human serum albumin (HSA)@indocyanine green–Bevacizumab (NPs‐Bev) is synthesized to improve the efficacy of fluorescence‐image‐guided BCS and RT. Fluorescence image guidance of the second near infrared NP improves complete resection in tumor‐bearing mice and accurately discriminates between benign and malignant mammary tissue in transgenic mice. Moreover, targeting tumors with NPs induces more reactive oxygen species under X‐ray radiation therapy, which not only increases RT sensitivity, but also reduces tumor progression in mice. Interestingly, self‐assembled NPs‐Bev using HSA, the magnetic resonance contrast agent and Bevacizumab‐targeting vascular growth factor A, which are clinically safe reagents, are safe in vitro and in vivo. Therefore, the novel self‐assembled NPs provide a solid precision therapy platform to treat breast cancer.

## Graphical abstract

This study synthesizes a novel self‐assembled multifunctional nanoprobe (NP) gadolinium–diethylenetriaminepentaacetic acid–human serum albumin@indocyanine green (ICG)–Bevacizumab to successfully improve the efficacy of fluorescence‐image‐guided surgery and radiotherapy for breast cancer (BC). Interestingly, the platform's components are all clinically safe reagents. In the future, this NP is expected to provide a precision treatment platform for BC.

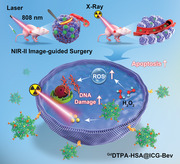

## 1 Introduction

Tumor‐free surgical margins are critical parameters for breast‐conserving surgery (BCS) as negative margins effectively reduce local tumor recurrence and distant metastasis, thereby optimizing clinical outcomes for patients.^[^ ^1^ ^]^ However, intraoperative tumor margin identification and sensitivity is poor as it mainly relies on palpation and visual inspection. Specifically, ≈20–40% of patients require further surgery or eventually undergo a mastectomy.^[^ ^2^ ^]^ Resectomies increase complication risks, potentially delay systemic treatments, and increase costs and health care burdens. Also, intraoperative margin evaluation approaches remain challenging for conventional imaging methods such as X‐ray radiography, magnetic resonance imaging (MRI), and computed tomography (CT), as sensitivity and specificity are often limited, and methods are difficult to apply in operating rooms.^[^ ^3^ ^]^ Thus, a real‐time, specific modality evaluating margins is urgently required in clinical settings.^[^ ^4^ ^]^ Recently, second near‐infrared (NIR‐II) window (1000–1700 nm) fluorescence imaging, characterized by high tumor‐to‐background ratios (TBRs) and deep tissue penetration, has become a promising strategy for accurate image‐guided tumor surgery. To date, NIR‐II contrast agents, including quantum dots, rare‐earth‐doped nanoparticles, single‐walled carbon nanotubes, and organic dyes have been developed.^[^ ^5^ ^]^ However, these reagents are not approved for clinical use. Intriguingly, clinically approved indocyanine green (ICG) produces long off‐peak NIR‐II emission spectra, which have high quantum yields (QYs) in off‐peak regions, and are particularly higher than most inorganic NIR‐II nanoprobes (NPs).^[^ ^6^ ^]^

Radiotherapy (RT) is a widely used cancer treatment modality for locally advanced breast cancer.^[^ ^7^ ^]^ However, clinical RT is limited^[^ ^8^ ^]^ and side effects are often observed due to RT‐induced damage to normal tissue.^[^ ^9^ ^]^ Therefore, to effectively overcome these defects, RT doses at tumor areas must be selectively increased which may improve treatment efficacy and reduce associated side effects. Gadolinium (Gd) exhibits dose‐dependent RT enhancement due to its high atomic number (*Z* = 64) and significant compton scattering effects under high intensity X‐ray irradiation. Thus, biosafe‐Gd nanosensitizers’ development and their use for RT in patients with advanced breast cancer is warranted.

Molecular‐image‐guided cancer therapy has become a highly promising research area as it significantly improves cancer therapy efficacy. Molecular‐image‐guided cancer therapy not only precisely localizes tumors, but also facilitates the continuous enrichment of therapeutic drugs in vivo. The quest for better molecular‐image‐guided cancer therapy has motivated the development of multifunctional drugs. To date, many inorganic nanocomposites have been reported, such as iron‐oxide‐based nanoscale,^[^ ^10^ ^]^ gold‐based,^[^ ^11^ ^]^ and transition‐metal‐disulfide‐based nanoparticles.^[^ ^12^ ^]^ However, most nanoplatforms are largely unsatisfactory in terms of biostability, biocompatibility, and preparation feasibility, which restrict their clinical application. Therefore, we strategically integrated the NIR dye ICG, clinically approved by the Food and Drug Administration (FDA), into ^Gd^DTPA–human serum albumin (HSA)@ICG NPs containing glycyrrhetinic‐acid‐modified Gd–diethylenetriaminepentaacetic acid (Gd–DTPA) to establish a multifunctional nanoreagent ^Gd^DTPA–HSA@ICG–Bevacizumab (NPs‐Bev), for MR, fluorescence imaging, and RT. NPs‐Bev had a TBR of >6 under NIR‐II imaging, and the relapse‐free survival rate was significantly improved (*p* = 0.0085). Also, probe relaxation rates were improved, and were used for the preoperative diagnosis of breast masses and RT sensitization. Our nanoplatform has several advantages: i) highly biocompatible with excellent imaging and therapeutic capabilities, ii) enhanced T1 contrast capabilities and NIR‐II fluorescence properties for precise imaging and guided surgery, and iii) good in vivo stability and easy to construct.

For the first time, we developed a highly biocompatible and feasible therapeutic reagent, which may become a promising multifunctional nanoplatform for the precision treatment of breast cancer in the future.

## 2 Results

### 2.1 NPs‐Bev Synthesis and Characterization

**Scheme 1:**
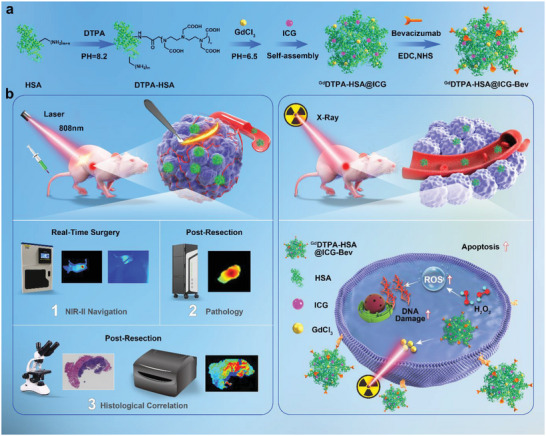
a) Schematic showing ^Gd^DTPA–HSA@ICG–Bevacizumab (NPs‐Bev) self­assembly formation using HSA, Gd–DTPA, ICG, and Bevacizumab. b) NPs‐Bev used for NIR‐II surgical navigation and radiotherapy sensitization.

**Figure 1:**
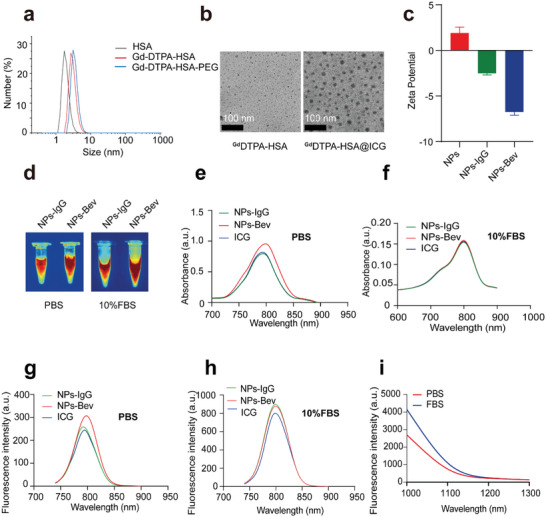
Characterization of NPs‐Bev and NPs‐IgG. a) Mass spectrometry analysis of HSA, Gd–DTPA–HSA, Gd–DTPA–HSA−PEG. b) TEM images showing ^Gd^DTPA–HSA (left) and ^Gd^DTPA–HSA@ICG (right). Scale bar = 100 nm. c) The zeta potentials of NPs, NPs‐IgG, and NPs‐Bev. d) Representative NIR‐II fluorescence image showing NPs‐IgG and NPs‐Bev in PBS or 10% FBS e,f) The absorption spectra of NPs‐Bev, NPs‐IgG, and ICG in PBS (e) and 10% FBS (f). g,h) Fluorescence emission spectra of NPs‐Bev, NPs‐IgG, and ICG in PBS (g) and 10% FBS (h). i) Fluorescence emission spectra of NPs‐Bev in PBS or 10% FBS under NIR‐II fluorescence excitation (Ex = 808 nm).

We explored the formation of self‐assembled nanocomposites using different input HSA, GdCl_3_, and ICG ratios. The input 1:5:2 (HSA:Gd^3+^:ICG) molar ratio was selected to fabricate NPs. As shown (**Scheme** **1**), DTPA groups were covalently bound to HSA amine moieties, and the resultant DTPA–HSA conjugate was characterized using matrix assited laser desorption ionization‐time of flight mass spectrometry (MALDI‐TOF MS) (Figure S1a, Supporting Information). An increase in molecular weight was observed, from 66.9 kDa (native HSA) to 72 kDa (DTPA–HSA). Also, Gd^3+^ was chelated to DTPA–HSA. Then, ICG, as a hydrophobic drug, was linked with ^Gd^DTPA–HSA molecules to form nanosized ^Gd^DTPA–HSA@ICG particles via self‐assembly. Using a particle size analyzer and transmission electron microscopy (TEM), NPs were 7–10 nm and spherical in shape (Figure 1a,b), which meant that HSA had successfully induced ^Gd^DTPA–HSA self‐assembly into nanoparticles. Zeta potential analyses showed that the ^Gd^DTPA–HSA@ICG–Immunoglobulin G (NPs‐IgG) potential (control tracer), which was NP conjugated to normal IgG, and NPs‐Bev went from positive to negative when compared with NPs (Figure 1c). NPs‐IgG and NPs‐Bev dissolved in phosphate buffered saline (PBS) or 10% fetal bovine serum (FBS) exhibited stable NIR‐II fluorescence signals (Figure 1d). Subsequently, sodium dodecyl sulfate–polyacrylamide gel electrophoresis showed that NIR fluorescence signals in NPs‐Bev and NPs‐IgG were consistent with gel protein positions, indicating that Bev or IgG had successfully conjugated to NPs (Figure S1b,c, Supporting Information). Furthermore, the fluorescence intensity of both probes was extremely stable after continuous observations for 96 h in PBS or 10% FBS (Figure S5a–f, Supporting Information). These characteristics provided important indications for the safe use of these NPs in organisms.

To investigate NP optical properties, the fluorescence signal intensities of ICG, NPs‐Bev, and NPs‐IgG molecules were measured in PBS (Figure 1e) or 10% FBS (Figure 1f). The absorption peak of ICG was 786 nm, NPs‐Bev was 790 nm, and NPs‐IgG 788 nm, indicating that absorption waves underwent infrared peak shifts. The emission waves of ICG, NPs‐Bev, and NPs‐IgG were measured in PBS (Figure 1g) or 10% FBS (Figure 1h). The emission peak of ICG was 795 nm, NPs‐Bev 796 nm, and NPs‐IgG 795 nm. Additionally, both probes also exhibited long trailing signals in the NIR‐II region (Figure 1i). To investigate ICG stability in NPs‐Bev, we dialyzed NPs‐Bev with PBS or PBS plus 10% FBS, with ICG aqueous solution as a control. ICG release was relatively low in PBS (Figure S2, Supporting Information). However, in the ICG group in 10% FBS, ≈88% ICG was released after 48 h. By contrast, the percentage ICG released by the NPs‐Bev group in 10% FBS was ≈22% after 48 h, and was probably due to ICG stably embedding into NPs‐Bev, with little leakage into the physiological environment.

NPs‐Bev and NPs‐IgG were dissolved in PBS at pH 7.4 (normal tissue) and 6.5 (tumor tissue) under visible light, with no significant changes observed after 48 h (Figure S3a, Supporting Information). To determine NPs‐Bev and NPs‐IgG stability at different pH conditions, NIR‐I fluorescence absorbance (Figure S3a–e, Supporting Information), NIR‐I fluorescence emission (Figure S4a–d, Supporting Information), and NIR‐II trailing studies were performed (Figure S4e,f, Supporting Information), and showed that the fluorescence properties of NPs‐Bev and NPs‐IgG were stable under different pH conditions.

To further analyze the NIR fluorescence properties of ICG, we simulated tissue in 1% intralipid solution to test ICG penetration depth in NIR‐I and NIR‐II devices. ICG penetrated up to 7 mm at the NIR‐II region in 1% intralipid solution, but <5 mm at the NIR‐I region (Figure S6a, Supporting Information). The fluorescence intensity signal of lower limb blood vessels in mice under NIR‐II region fluorescence imaging was ≈2.3 times when compared with the NIR‐I region (Figure S6b–e, Supporting Information). Thus, NPs‐Bev showed better contrast traits in the NIR‐II region and reached deeper penetration depths.

### 2.2 NPs‐Bev Cell Uptake

**Figure 2:**
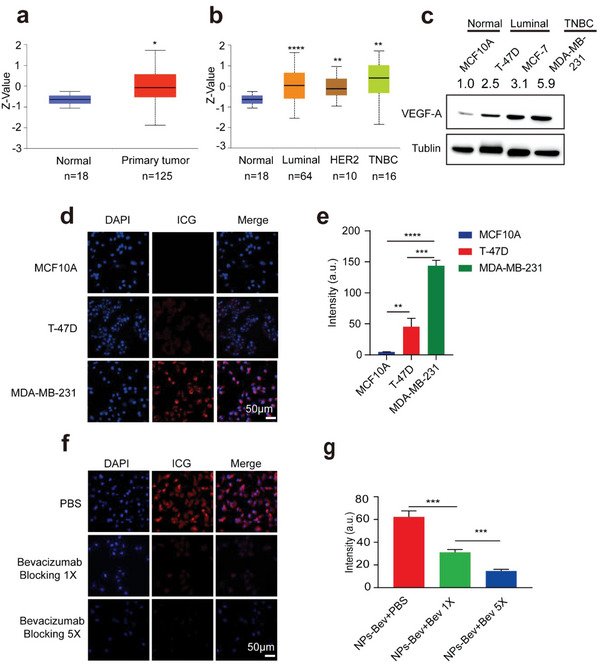
NPs‐Bev targets VEGF‐A in triple‐negative breast cancer cells. a) CPTAC public database samples (http://ualcan.path.uab.edu/index.html) showed that VEGF‐A protein expression levels in breast cancer patients were higher when compared with normal breast tissue (*p* < 0.01). b) VEGF‐A expression levels were the highest in triple‐negative breast cancer cells when compared with other subtypes. c) VEGF‐A expression in representative subtypes, T‐47D and MCF‐7 (luminal), MDA‐MB‐231 (TNBC), and MCF‐10A represented normal mammary epithelial cells. d) Confocal fluorescence analysis of different VEGF‐A expression levels in various breast cancer cell lines, MDA‐MB‐231, T‐47D, and MCF10A cells after NPs‐Bev (8 µg mL^−1^) was cocultured with cells for 4 h. e) Quantitative analysis of cell fluorescence signals (ImageJ software) showing that probe uptake was the highest in MDA‐MB‐231 cells when compared with T‐47D and MCF10A cells (*p* < 0.001). f) Confocal fluorescence analysis in MDA‐MB‐231 cells blocked with bevacizumab (250 and 1250 µg mL^−1^) at 30 min before coincubation with NPs‐Bev (8 µg mL^−1^ ICG). g) Fluorescent intensity decreased in NPs‐Bev + Bev (250 µg mL^−1^) when compared with PBS (31.07 ± 2.51 vs 62.22 ± 5.35, *p* = 0.0008) and NPs‐Bev + Bev (1250 µg mL^−1^) when compared with PBS (14.71 ± 1.46 vs 62.22 ± 5.35, *p* = 0.0001) in MDA‐MB‐231 cells. Scale bar = 50 µm.*p < 0.05, **p < 0.01, ***p < 0.001, ****p < 0.0001.

To clarify vascular growth factor A (VEGF‐A) expression levels in breast cancer patients, we searched the Clinical Proteomic Tumor Analysis Consortium (CPTAC) sample database (http://ualcan.path.uab.edu/index.html) and observed that VEGF‐A expression levels in breast cancer patients were higher when compared with normal breast tissue (*p* < 0.01) (**Figure** **2**a). Levels were the highest in triple‐negative breast cancer (TNBC) MDA‐MB‐231 cells when compared with other subtypes (Figure 2b). These data were consistent with VEGF‐A protein levels in our breast cancer cell lines (Figure 2c).

To identify optimal imaging times and concentrations, NPs‐Bev and NPs‐IgG molecules were cocultured with MDA‐MB‐231 cells at different concentrations (1, 2, 4, 8 µg mL^−1^, ICG content is quantitative) and incubation times (0.5, 1, 2, and 4 h). Quantitative fluorescence intensity analyses showed that probe uptake was higher for 8 µg mL^−1^ NPs‐Bev when compared with NPs‐IgG (115.99 ± 6.25 vs 33.52 ± 1.08; *p* < 0.0001), while coculturing for 4 h, NPs‐Bev levels were higher when compared with NPs‐IgG (214.68 ± 7.90 vs 95.32 ± 0.91; *p* < 0.0001) (Figure S7a–d, Supporting Information). Also, to verify cell targeting capabilities for different VEGF‐A expression levels in various breast cancer cell lines, MDA‐MB‐231, T‐47D, and MCF10A cells, NPs‐Bev (8 µg mL^−1^) was cocultured with these cell lines for 4 h. Quantitative cell fluorescence analyses showed that probe uptake was the highest in MDA‐MB‐231 cells (*p* < 0.001) (Figure 2d,e).

To further clarify probe targeting, competitive blocking studies were performed. PBS or free‐Bevacizumabbev antibody (250 or 1250 µg mL^−1^) was added to MDA‐MB‐231 culture medium 30 min before coincubation with NPs‐Bev (8 µg mL^−1^ ICG). Fluorescent intensity decreased in the NPs‐Bev + Bev (250 µg mL^−1^) group when compared with the PBS (31.07 ± 2.51 vs 62.22 ± 5.35, *p* = 0.0008) group, and the NPs‐Bev + Bev (1250 µg mL^−1^) group when compared with the PBS group (14.71 ± 1.46 vs 62.22 ± 5.35, *p* = 0.0001), indicating that NPs‐Bev specifically bound to MDA‐MB‐231 cells (Figure 2f,g).

### 2.3 Fluorescence Targeting Tumors and Various Microtumor Models In Vivo

**Figure 3:**
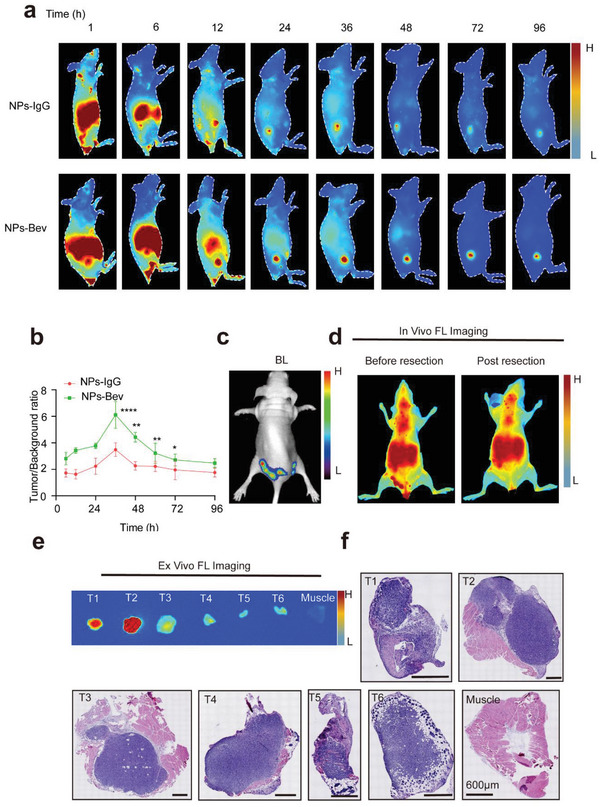
Targeted recognition of tumor and multiple microtumor models in vivo. a) NIR‐II fluorescence imaging in the NPs‐Bev group showed strong fluorescence signals in the tumor at 36 h postinjection. b) The maximum tumor‐to‐background ratio in the group at 36 h postinjection (6.77 ± 0.45) was higher when compared with the NPs‐IgG group at 36 h postinjection (2.48 ± 0.52). c) Preoperative bioluminescent (BL) images at preoperation in a representative multi‐microtumor mouse model. d) NPs‐Bev was injected preoperatively and postoperatively, and fluorescence images taken before and after tumor resection. e) In vitro fluorescence and f) the corresponding H&E‐stained histological images of tumor tissue (T1–T6) and muscle. Scale bar = 600 µm.*p < 0.05, **p < 0.01, ****p < 0.0001.

To determine the optimal imaging concentration and time, we performed continuous imaging observations according to ICG quantification, and observed that 2 mg kg^−1^ and injection at 36 h were optimal imaging concentration and time parameters (Figure S9a,b, Supporting Information). To further verify NPs‐Bev tumor‐targeting specificity in vivo, MDA‐MB‐231 tumor‐bearing mice were injected with NPs‐Bev and NPs‐IgG through the tail vein. NIR‐II fluorescence imaging showed that the NPs‐Bev group exhibited strong fluorescence in the tumor. Furthermore, the maximum TBR in the NPs‐Bev group (6.77 ± 0.45) was higher when compared with the NPs‐IgG group at 36 h postinjection (2.48 ± 0.52) (*p* = 0.0004), indicating that the NPs‐Bev probe targeted MDA‐MB‐231 tumor‐bearing mice (**Figure** **3**a,b). To determine NP biodistribution levels in vivo, the main organs from mice underwent NIR‐II fluorescence imaging at 36 h postinjection (Figure S9c,d, Supporting Information). Subsequently, Bev blocking studies were performed in vivo. MDA‐MB‐231‐Luc bearing mice were randomly divided into two groups (*n* = 3) and injected with PBS or Bev (250 mg kg^−1^) 30 min before NPs‐Bev injection (ICG = 2.0 mg kg^−1^). After bevacizumab blockade, the tumor aggregation of the probe was significantly inhibited at 12 h (*p* < 0.05) (Figure S10a,b, Supporting Information).

To test probe sensitivity, a multiple microtumor model was constructed to examine if fluorescent NPs could real‐time, accurately identify small tumors. We observed that real‐time tumor fluorescence and bioluminescence imaging signals were highly consistent (Figure 3b,c), and the corresponding tissues, which were pathologically resected by NIR‐II fluorescence guidance (Figure 3d), confirmed the entire boundary between the tumor and normal tissue (Figure 3e,f).

### 2.4 In Vitro and In Vivo NPs‐Bev MRI

**Figure 4:**
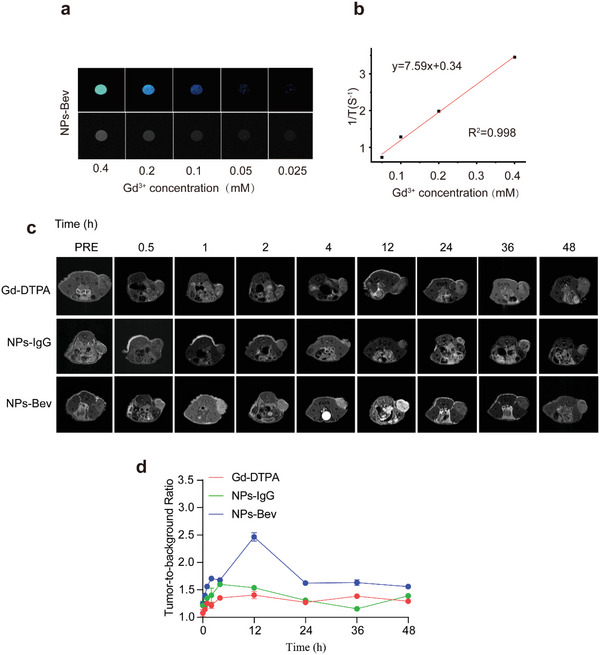
In vitro and in vivo MRI of NPs‐Bev. a) T1­weighted MR phantom images of NPs‐Bev and b) longitudinal relaxivities (r1) for NPs‐Bev using a 0.5T MRI scanner. c) Comparing relative maximum tumor background signal intensities in mice injected with NPs‐IgG, NPs‐Bev, and Gd–DTPA (*n* = 3). d) Tumor‐to‐background MRI ratio of mice intravenously injected with NPs‐IgG, NPs‐Bev, and Gd–DTPA ([Gd^3+^] = 25 × 10^−6^ m kg^−1^) at different times (*n* = 3).

Gd has five unpaired 3d electrons and may be used as a T1‐shortening agent for MRI.^[^ ^9^ ^]^ Thus, we compared the clinical contrast agents Gd–DTPA, NPs‐Bev, and NPs‐IgG MRI performances in vitro. Our results showed that relaxation degrees were 7.59 and 7.50, respectively, according to curve slopes between 1/T1 and the Gd concentration in a 0.5 T magnetic field (**Figure** **4**a,b and Figure S8a,b (Supporting Information)).

We explored in vivo MRI studies on MDA‐MB‐231 subcutaneous tumor mice using NPs‐Bev with relative stability of T relaxation rate. T1‐weighted images at different times (0.5, 1, 2, 4, 12, 24, 36, and 48 h) were collected using a 9.4T MRI scanner (Figure 4d). The TBR of the NPs‐Bev group was the highest at 2.465 ± 0.08, while NPs‐IgG was 1.54 ± 0.03, and Gd–DTPA was 1.41 ± 0.07 at 12 h after probe injection (*p* < 0.0001). (Figure 4c). These results confirmed that NPs‐Bev enhanced tumor contrast signals in T1‐weighted MR images.

### 2.5 NIR‐II‐Fluorescence‐Image‐Guided Surgery in MDA‐MB‐231‐Luc Tumor‐Bearing Mice

**Figure 5:**
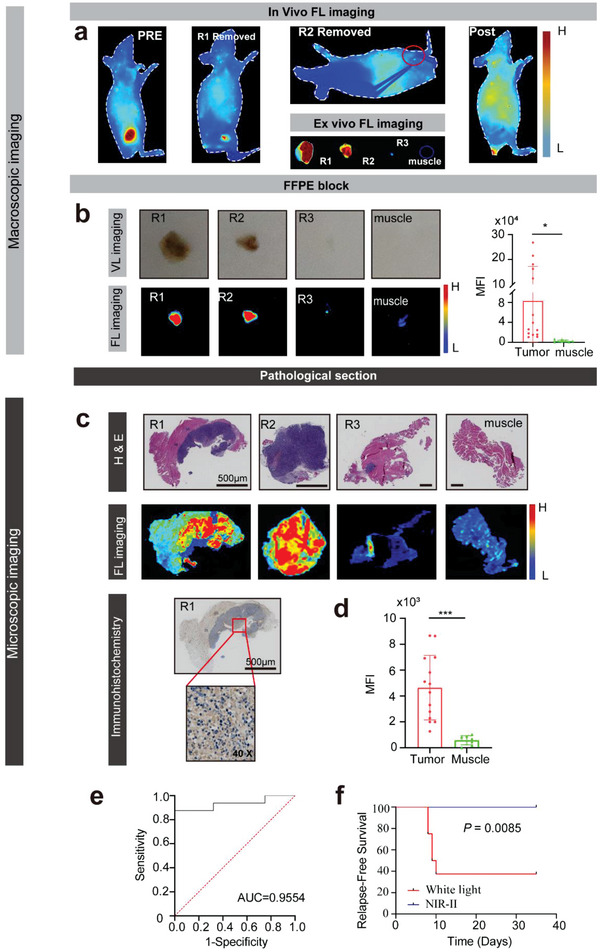
NIR‐II‐fluorescence‐image‐guided surgery in MDA‐MB‐231‐luc tumor‐bearing mice. a) Fluorescence (FL) imaging demonstrated dynamic tumor resection in three fluorescent‐guided surgeries (R1–R3) in MDA‐MB‐231‐luc tumor‐bearing mice at 36 h after injection of NPs‐Bev. b) FFPE block in visible light, FL imaging, and quantified MFI tumor, and margin (muscle) signal ratio. c) H&E‐stained histological images of surgical margins showing the primary tumor (R1–R3) and negative surgical margin (muscle) after three rounds of fluorescence‐guided surgery (first panel). 10 µm sections were scanned by fluorescence imaging (middle panel) and 4 µm sections underwent VEGF‐A immunohistochemical staining (last panel). d) Quantified MFI tumor and margin (muscle) signal ratios. e) Fluorescence imaging receiver operating characteristic (ROC) curve differentiating between normal tissue and cancer tissue. The AUC = 0.9554 (95% confidence interval [CI]: 0.836–1.0). f) Kaplan–Meier analysis showing significantly better survival in NIR‐II‐guided surgery groups when compared with white light only groups (*n* = 8, statistical significance was assessed using log‐rank tests, *p* = 0.0085). Scale bar = 500 µm.*p < 0.05, ***p < 0.001.

NIR‐II‐fluorescence‐image‐guided tumor surgery was performed at 36 h postinjection since the highest TBR was confirmed at this time. Furthermore, to simulate intraoperative tumor surgery, we evaluated fluorescence‐image‐guided surgery feasibility in a MDA‐MB‐231‐luc tumor‐bearing mouse model. For surgical resection, NPs‐Bev molecules were injected into mice. As shown (**Figure** **5**), the NIR‐II navigation surgical group was intravenously injected with NPs‐Bev (ICG dose = 2.0 mg kg^−1^) and tumors were resected at 36 h postinjection. We then completely removed the tumor under NIR‐II surgical navigator guidance, and observed that the smallest recognizable residual tumor was ≈0.5 mm (Figure 5a). The tumor and adjacent muscle tissue were excised, formalin‐fixed and paraffin‐embedded (FFPE) into blocks (Figure 5b), and sectioned into 10 µm slices for fluorescence imaging and 4 µm slices for hematoxylin and eosin (H&E) staining and VEGF‐A immunohistochemistry (IHC). To compare fluorescence (FL) signals between tumor and muscle tissue in FFPE and 10 µm sections, FL was captured, and the tumor's FL mean ratio was nearly 40 times when compared with muscle levels (*p* = 0.020) in FFPE block, and the signal was about 8 times stronger than that of muscle in 10 µm sections (Figure 5c,d). The area under the curve (AUC) based on fluorescence assessment between malignant and benign tissue was 0.9554 (95% confidence interval (CI): 0.836–1.0) (Figure 5e). NIR‐II‐fluorescence‐based tumor tissue identification was confirmed by H&E. Finally, the residual tumor was evaluated by bioluminescence imaging at day 35. In the NPs‐Bev group, no mice (0/8, 0%) exhibited residual tumor bioluminescence signals. However, 5/8 mice (62.5%) postoperatively presented with residual tumor signals in the white light group which performed the operation under white light (*p* = 0.0085) (Figure 5f and Figure S11 (Supporting Information)), indicating that NPs‐Bev showed excellent performance as a NIR‐II fluorescence agent.

### 2.6 NIR‐II Fluorescence Surgical Imaging in Spontaneous Breast Cancer Mice

**Figure 6:**
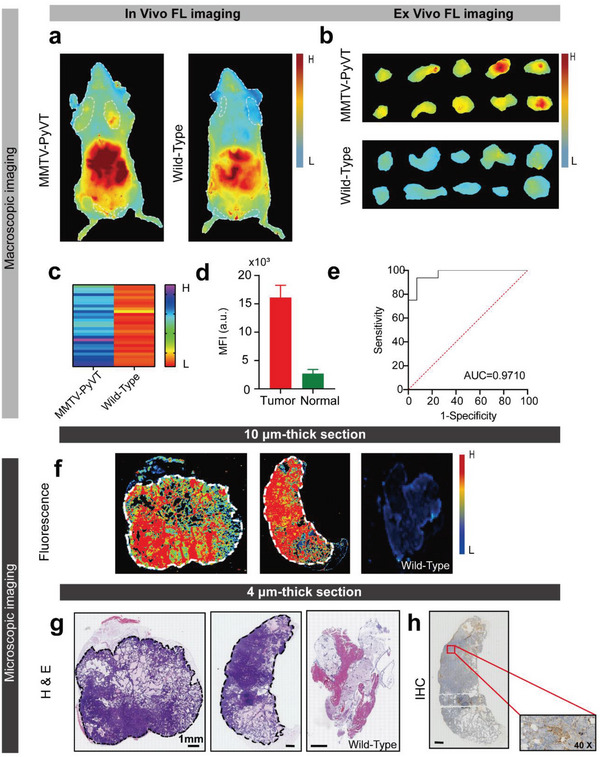
NIR‐II fluorescence imaging in a spontaneous breast cancer mouse model. a) In vivo and b) ex vivo fluorescence images showing mammary glands at 36 h after MMTV‐PyVT transgenic and wild‐type mice were intravenously injected with NPs‐Bev. c) Mean fluorescence intensities (MFIs) of 1st–5th resected mammary gland tissue layers in MMTV‐PyVT transgenic and wild‐type mice (*n* = 30) ****p < 0.0001. d) MFI was detected in 10 µm thick transgenic mouse tumor tissue or healthy breast tissue by Odyssey imaging system. e) Fluorescence imaging ROC curve differentiating between normal tissue and cancer tumors. AUC = 0.9710 (95% confidence interval [CI]: 0.9294–1.0) (*n* = 3). f) Microscopic biodistribution of NPs‐Bev in breast tissue. The upper row shows fluorescence images of 10 µm mouse breast tissue slices. The lower row shows the corresponding H&E staining. g) Representative breast tissue sample shows a H&E section (middle) and h) immunohistochemical VEGF‐A staining. Scale bar = 1 mm.

To further simulate human breast cancer development, NPs‐Bev molecules were explored as NIR‐II fluorescence agents in fluorescence‐imaging‐guided surgery in spontaneous breast cancer mice. In vivo and ex vivo fluorescence imaging of Mouse Mammary Tumor Virus‐Polyomavirus middle T antigen (MMTV‐PyVT) mice injected with NPs‐Bev through the tail vein showed highly intense fluorescence signals in the mammary gland, whereas normal breast tissue signals in wild‐type mice were extremely weak (**Figure** **6**a–d). Subsequent fluorescence analysis showed that the breast tumor exhibited a higher fluorescence intensity when compared with the normal breast, and the AUC for fluorescence discrimination between malignant and benign tissue was 0.9710 (95% CI: 0.9294–1.0; *p* < 0.01), suggesting that NPs‐Bev‐based NIR‐II fluorescence imaging accurately distinguished tumors from healthy tissue during surgery (Figure 6e). The NIR fluorescence imaging system was used to scan mammary tissue slices and analyze NP distributions in mammary tissue. Conspicuously, fluorescence signals in microscopic tumors had higher intensities when compared with normal breast tissue (Figure 6f). All sections were stained with H&E and analyzed by IHC. As shown (Figure 6g), microscopic NPs‐Bev fluorescence images clearly distinguished invasive carcinoma from healthy glands, consistent with H&E staining and VEGF‐A IHC (Figure 6h), and clearly demonstrated that NPs accurately identified tumor properties.

### 2.7 Radiation Sensitization in Cells

**Figure 7:**
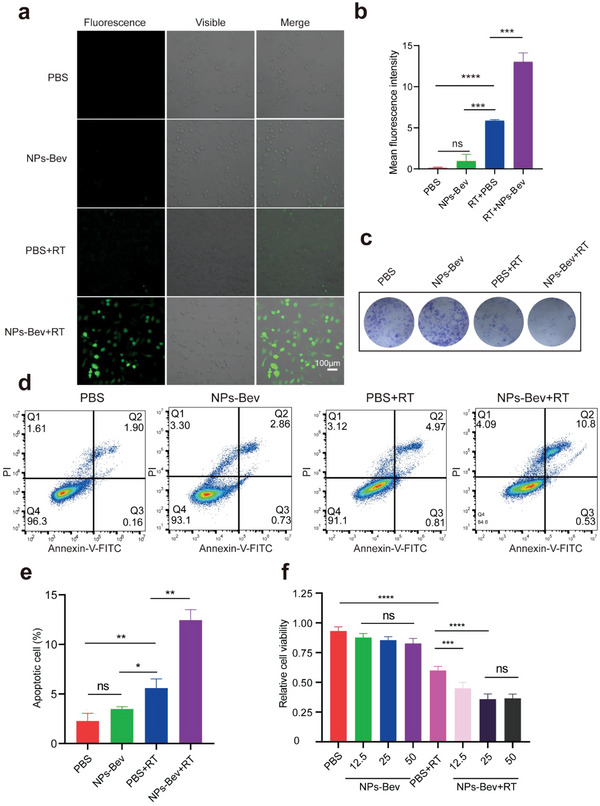
In vitro NPs‐Bev RT sensitization. a) Confocal laser‐scanning microscopy images showing intracellular ROS levels using a ROS probe (H2DCFDA) in MDA‐MB‐231 cells treated with PBS and NPs‐Bev, with or without X‐ray irradiation (6 Gy). b) Quantification of MFI from confocal laser‐scanning microscope images using ImageJ software (*n* = 3). c) Colony formation assays of MDA‐MB‐231 cells treated with PBS and NPs‐Bev, with or without X‐ray irradiation (6 Gy). d) Flow cytometry analysis and e) quantified apoptosis rates in MDA‐MB‐231 cells treated with PBS and NPs‐Bev under X‐ray (6 Gy) irradiation. Nonirradiated PBS and NPs‐Bev were used as the control group (*n* = 3). f) Cell counting assays showing MDA‐MB‐231 cells treated with PBS and different concentrations ([Gd^3+^] = 12.5 × 10^−6^, 25 × 10^−6^, and 50 × 10^−6^ m) of NPs‐Bev under X‐ray (6 Gy) irradiation. Nonirradiated PBS and NPs‐Bev were used as controls (*n* = 5). Scale bar = 100 µm.*p < 0.05, **p < 0.01, ***p < 0.001, ****p < 0.0001, ns: not statistically significant.

To evaluate NPs‐Bev sensitization by RT, we used 2ʹ,7ʹ‐dichlorodihydrofluorescein diacetate (H2DCFDA) to monitor intracellular reactive oxygen species (ROS) levels.^[^ ^13^ ^]^ Using X‐ray irradiation (6 Gy), intracellular fluorescence in MDA‐MB‐231 cells incubated with NPs‐Bev was significantly stronger when compared with PBS‐incubated cells (*p =* 0.0004). Also, fluorescence signals from nonirradiated PBS and NPs‐Bev groups were negligible (**Figure** **7**a,b). Thus, NPs‐Bev significantly increased intracellular ROS production under X‐ray irradiation. Moreover, colony formation assays were performed to evaluate long‐term NPs‐Bev radiation sensitization (Figure 7c). X‐ray‐irradiated MDA‐MB‐231 tumor cells showed a small number of viable cell colonies when incubated with nanoparticles, significantly less than the radiation group (Figure S12, Supporting Information). The effects of NPs‐Bev combined with RT on apoptosis were also analyzed. Flow cytometry showed that apoptosis in the NPs‐Bev plus RT group was significantly higher when compared with the PBS plus RT group (*p =* 0.0011) (Figure 7d,e).

We also determined cytotoxicity levels of different NPs‐Bev concentrations toward MDA‐MB‐231 cells. Gd^3+^ concentrations up to 50 × 10^−6^ m exerted no obvious cytotoxicity effects in tumor cells, indicating excellent nanoparticle biocompatibility. However, under X‐ray irradiation, nanoparticle cytotoxicity (Gd^3+^ concentration = 12.5 × 10^−6^ m) was significantly higher when compared with radiation alone, indicating that RT mediated by nanoparticles greatly improved radiation killing abilities toward tumor cells (*p* < 0.0001) (Figure 7f).

### 2.8 In Vivo Radiosensitization Studies

**Figure 8:**
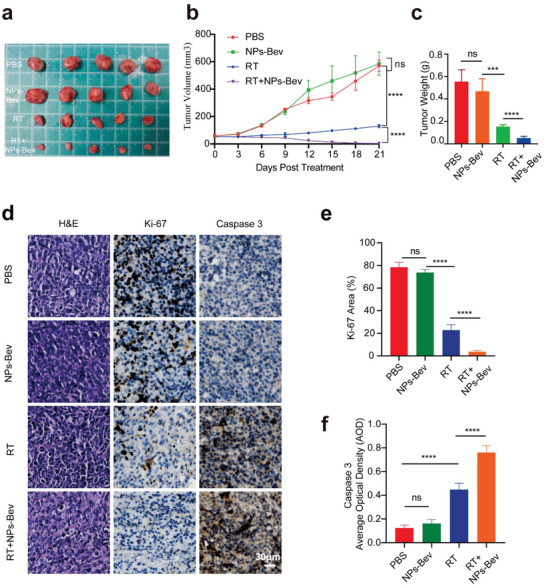
Radiosensitization of NPs‐Bev in MDA‐MB‐231 tumor‐bearing mice. a) In vitro tumors represent mice treated with NPs‐Bev and PBS with or without X‐ray RT (*n* = 5). b) Tumor growth curves of mice treated with NPs‐Bev and PBS, with or without X‐ray RT (*n* = 5). RT was conducted on days 0 and 6, and each exposure was 6 Gy. c) Tumor weight after different treatments at day 21 (*n* = 5). d) H&E staining and Ki‐67 and Caspase 3 IHC staining in tumor slices after different treatments. The corresponding quantitative analyses of e) Ki‐67 and f) Caspase 3. Scale bar = 30 µm.***p < 0.01, ****p < 0.0001, ns: not statistically significant.

Owing to Gd compton scattering under X‐ray irradiation, we evaluated tumor elimination efficiencies in mice injected intravenously with NPs‐Bev. Representative fluorescence images in mice before treatment with NPs‐Bev and PBS, with or without X‐ray RT, are shown (Figure S13, Supporting Information), which demonstrated that the novel probe could specifically target breast tumors, and we subsequently performed tumor radiotherapy. As shown (**Figure** **8**a,b), nonirradiated NPs‐Bev‐treated mice showed almost no inhibited tumor growth. However, under X‐ray irradiation, NPs‐Bev‐treated mice showed effective RT sensitization and significant tumor regression when compared with mice treated with X‐ray radiation alone. Additionally, weight assessments of resected tumors at day 21 confirmed significant tumor eradication and tumor growth inhibition in RT‐sensitized animals when compared with the other groups (Figure 8c). There were no differences in body weight between the four groups PBS, NPs‐Bev, RT, and RT + NPs‐Bev (Figure S14, Supporting Information). Furthermore, the antitumor effectiveness in different tumor tissues was analyzed by IHC. Tumor sections stained with the Ki‐67 marker indicated that in mice with NPs‐Bev combined with RT, animals showed fewer hyperproliferative tumor cells when compared with the other groups (Figure 8d,e). Additionally, caspase‐3 activity was significantly increased in NPs‐Bev combined with X‐ray radiation animals when compared with the other groups (Figure 8f). Therefore, NPs‐Bev + RT treatments activated caspase‐3, possibly by inhibiting proliferation and inducing apoptosis in cancer cells.

### 2.9 NPs‐Bev Biosafety In Vivo and Ex Vivo

Cell counting kit‐8 assays were used to examine NPs‐IgG and NPs‐Bev cytotoxicity in MDA‐MB‐231, T‐47D, and MCF‐10A cells. No significant cell viability inhibition was observed with increasing probe concentrations (1, 10, 50, and 100 µg mL^−1^) for 72 h (Figure S15, Supporting Information), thereby indicating that probes did not affect cell viability and were suitable for imaging in vivo.

To verify in vivo safety, mice were randomly divided into two groups after the tail vein injection of NPs‐Bev and PBS. H&E‐stained organs showed no obvious pathological changes in both groups (Figure S16a, Supporting Information). Thus, NPs‐Bev toxicity in mice was negligible and showed that probes could be safely used in future clinical trials. Venous blood was also collected for liver and kidney functions or routine blood analysis on days 1, 3, 7, and 28. The results showed that alanine aminotransferase (ALT), aspartate aminotransferase (AST), creatine kinase MB (CK‐MB), UREA, and creatinine (CREA) levels showed no significant changes (Figure S16b,c, Supporting Information). Additionally, no significant differences were observed in white blood cell (WBC), lymphocyte (Lymph), monocyte cell (Mon#), neutrophil granulocyte (Gran#), red blood cell (RBC), hemoglobin (HGB), and platelet (PLT) levels. We also observed no differences in body weight between groups, indicating that NPs‐Bev‐based RT sensitization caused no cytotoxicity in vivo (Figure S16d, Supporting Information).

## 3 Discussion

Positive margin is highly associated with poor tumor localization accuracy and inaccurate tumor removal through viewing examination and touch‐based feedback. It is now recognized that obtaining a clean margin during the first surgery remains an important physical behavior in BCS.^[^ ^14^ ^]^ Imaging‐guided surgery is becoming more important in clinical settings. It helps surgeons identify small‐sized diseased growths or leftover wounds which are easily missed during surgery, and guide intraoperative surgical margin in addition assessment.^[^ ^15^ ^]^ Therefore, this approach may improve prognoses in patients undergoing cancer surgery.

Molecular NIR fluorescence is a novel imaging modality with several distinct advantages, including no ionizing radiation and visualization when compared with positron emission tomography, CT, and other imaging methods.^[^ ^16^ ^]^ In 2013, we successfully used ICG to surgically navigate a sentinel lymph node biopsy in breast cancer.^[^ ^17^ ^]^ Keating et al.^[^ ^18^ ^]^ used the Artemis NIR imaging device combined with ICG to guide BCS, and observed that half of patients had residual fluorescence signals in the tumor bed after tumor removal (6/12), but final pathological examinations confirmed negative margins. Therefore, this simple dye method generates high false positive rates in evaluating BCS margin status. Intriguingly, the development of tumor‐specific targeting molecular probes has accelerated optical molecular imaging for the accurate determination of surgical margins in BCS.^[^ ^19^ ^]^ For example, monoclonal antibodies, such as Bev and cetuximab have been developed to target VEGF‐A and epidermal growth factor receptor, which are characteristic tumor cell surface markers.

Indeed, using FDA‐approved antibodies to target NIR dye distribution is highly favorable as pharmacokinetic and pharmacodynamic properties are already known from drug approval studies, thereby making it easier to customize tracers for clinical use.^[^ ^20^ ^]^ In previous research, we synthesized the NIR tracer Bev‐800CW which targeted VEGF‐A at high doses at both macro‐ and microlevels, and observed an 88% increase in the intraoperative detection of tumor margins.^[^ ^19^ ^]^ However, due to absorption and scattering effects of water molecules, organic biomolecules, other substances on the band, and autofluorescence from biological tissues, the depth and resolution of NIR‐I fluorescence imaging remains limited in clinical applications.^[^ ^21^ ^]^ Of note, NIR‐II fluorescence imaging significantly reduced spontaneous fluorescence and photon absorption and scattering in biological tissue, and generated high resolution and penetration.^[^ ^22^ ^]^ Many NIR‐II fluorescent materials have been developed, including small molecular organic dyes,^[^ ^23^ ^]^ carbon nanotubes,^[^ ^24^ ^]^ quantum dots,^[^ ^25^ ^]^ and rare earth materials,^[^ ^26^ ^]^ for use in biomedicine. Unfortunately, due to poor biocompatibility and unstable optical properties, the clinical application of these materials is restricted. Recently, FDA‐approved ICG was used in NIR‐II imaging as its QY was higher when compared with most synthetic NIR‐II emission contrast agents.^[^ ^27^ ^]^ In our study, ICG penetrated up to 7 mm in the NIR‐II region in 1% intralipid solution, but less in the NIR‐I region (5 mm). Moreover, fluorescence intensity signals in mice in the fluorescence NIR‐II imaging region were over 2 times greater when compared with the NIR‐I region. Therefore, ICG facilitated the clinical application of NIR‐II fluorescence imaging in vivo.

Suo et al.^[^ ^28^ ^]^ synthesized a Bev–ICG NIR‐II probe which targeted VEGF‐A in a rat colorectal cancer model, and showed that probe injection and NIR‐II fluorescence endoscopy guidance identified tumors that were previously difficult to find under white light conditions. In our study, NPs‐Bev showed superior performance as a NIR‐II fluorescent contrast agent, with fewer postoperative tumor recurrences (0/8, 0%) when compared with the white light group. We also constructed a multiple small tumor model and showed the probe accurately identified small tumors. We then used the spontaneous breast cancer transgenic mouse model^[^ ^14^ ^]^ to simulate breast cancer growth patterns, and showed that NPs‐Bev accurately distinguished between cancerous and healthy glandular tissue, achieving an AUC = 0.9710. NP‐based NIR‐II fluorescence image guidance improved complete tumor resection and relapse‐free survival rates and accurately distinguished between benign and malignant breast tissue.

As Gd^3+^ functions as a MRI contrast agent, commercial MRI contrast agents such as Gd^3+^ chelate and Gd–DTPA were first approved by the FDA for clinical use.^[^ ^29^ ^]^ Unfortunately, due to poor targeting and a short cycle life, Gd–DTPA is difficult to target to tumors as weak signals are generated.^[^ ^30^ ^]^ Encapsulating discrete Gd^3+^ chelates into nanoassembled capsules is a simple and effective way to prepare MRI contrast agents, and generates high relaxation imaging agents capable of carrying large loads.^[^ ^31^ ^]^ We previously reported that nanostructured Gd had good relaxation properties and generated high tumor MRI imaging signals.^[^ ^32^ ^]^ Consistent with these results, we confirmed that NPs‐Bev enhanced tumor contrast signals in T1‐weighted MR images.

Importantly, effectively increasing energy deposition in tumor areas or improving tumor targeting may improve tumor radiation therapy efficacy and reduce side effects associated with this therapy.^[^ ^33^ ^]^ In the last decade, several nanomaterials were developed and tested as radiosensitizers, of which high *Z* nanoparticles (HZNPs) attracted considerable research attention. Radiosensitization effects were observed in HZNPs constructed of gold, silver, bismuth, and Gd. Of note, motexafin Gd demonstrated good clinical benefits and rendered tumor cells more sensitive to RT (ClinicalTrials.gov Identifier: NCT00003411). Another highly anticipated reagent is activation and guidance of irradiation by X‐ray (AGuIX) nanoparticles, novel Gd chelate nanoparticles which have shown enhanced RT efficacy in multiple clinical trials.^[^ ^34^ ^]^ In our previous study, gadolinium oxide rare earth particles were nanosized to reduce Gd nanoparticle toxicity and degradation in the acidic tumor microenvironment to facilitate rapid in vivo metabolism for BCS and radiotherapy sensitization.^[^ ^9^ ^]^ While research results are promising, nanoparticles with aforementioned inorganic structures have issues such as uncontrolled metal leakage and heavy metal toxicity.^[^ ^35^ ^]^ In recent years, nanomaterial engineering research has opened up diagnosis and treatment avenues for the treatment of different diseases.^[^ ^36^ ^]^ In particular, self‐assembled organic nanomaterials have fine structures, convenient processing, low costs, good biocompatibility, enhanced permeability and retention effects, multifunctional properties, and outstanding application potential in the biomedical field.^[^ ^37^ ^]^ HSA is a natural biological macromolecule with good biocompatibility and low immunogenicity;^[^ ^38^ ^]^ it has been used as a carrier for the antitumor drug paclitaxel and is widely used in clinical settings.^[^ ^39^ ^]^ More importantly, Gd exhibits high hydrophobicity and may be used as an “adhesive” component between adjacent albumins to induce albumin assembly to form large nanoparticles. The process is similar to FDA‐approved paclitaxel–albumin nanoparticles (Abraxane),^[^ ^40^ ^]^ and may be a viable way to reduce the potential toxicity of Gd nanoparticles, improve biocompatibility, and avoid endoplasmic–reticuloendothelial system deposition.

We developed a novel self‐assembled multifunctional NP (NPs‐Bev) to integrate RT sensitization and facilitate real‐time efficacy. In NPs‐Bev‐treated mice, effective RT sensitization and significant tumor regression was generated under X‐ray irradiation when compared with mice treated with X‐ray radiation alone. Additionally, preliminary toxicological studies demonstrated good NP tolerability, and no significant pathological changes were observed in main mice organs. Therefore, our NP showed good biosafety performance.

In summary, we constructed a multifunctional molecular imaging system based on the self‐assembly pharmaceutical adjunct HSA, Gd–DTPA, and Bev to provide a research platform for the accurate navigation of BCS and RT sensitization. Recently, Rosenthal et al.^[^ ^41^ ^]^ designed a roadmap to regulate the development, formulation, current good manufacturing practice, and translation of fluorescent tracers. We aim to promote the clinical progress of molecular probes in China, and develop more multifunctional probes based on this roadmap. We anticipate that our work will provide accurate diagnosis and treatment outcomes for breast cancer in the future.

## 4 Experimental Section

### Cell Culture

Human breast cancer cell lines MDA‐MB‐231, MDA‐MB‐231‐luc, MCF‐7, T‐47D, MCF‐10A(normal epithelial cells) were obtained from Procell Life Science & Technology Co., Ltd. (Wuhan, China). Cells were cultured according to vendor recommendations.

### Animals

Animal studies followed strict guidance from the Institutional Animal Care and Use Committee of Xiamen University Animal Studies Committee. 4–6 weeks old female BALB/c nude mice were purchased from the Experimental Animal Center, Xiamen University. FVB/N‐Tg (MMTV PyVT) 634Mul/J transgenic mice were purchased from the Jackson Laboratory, USA.

### NPs‐Bev and IgG‐NP Synthesis

2 g HSA (Beyotime Biotechnology, China) was dissolved in 30 mL 0.1 m NaHCO_3_ (pH 8.2). Then, 2 g diethylenetriaminepentaacetic acid dianhydride was dissolved in 10 mL dimethyl sulfoxide (DMSO) and added to the HSA solution. The pH was adjusted to 8.2 using 1 m NaOH. The solution was stirred for 2 h at room temperature and dialyzed against deionized water. Then, 1 g GdCl_3_ (J&K Scientific, China) was added at a pH of 6.5 to generate ^Gd^DTPA–HSA. Mass spectrometry was then conducted to verify the Gd linking efficiency. Then, 60 mg ^Gd^DTPA–HSA solution was mixed with free ICG (J&K Scientific, China) (10 mg dissolved in 2 mL DMSO). The solution was stirred for 12 h at room temperature. Finally, the mixture was purified using a Sephadex G50 column (GE 121 Healthcare, USA).

Next, 25 mg ^Gd^DTPA–HSA@ICG nanoparticles were linked to 2 mg Bev antibodies (Roche Pharma, Switzerland) using *N*‐hydroxy‐succinimide (NHS)—polyethylene glycol (PEG) 2000—COOH (Aladdin, China). The carboxylic group was activated by *N*‐(3‐dimethylaminopropyl)‐*N′*‐ethylcarbodiimide hydrochloride (EDC•HCl) and NHS (Energy Chemical, Shanghai) to generate a fluorescence/magnetic resonance dual‐mode functionalized tumor‐targeting VEGF‐A multifunctional probe, NPs‐Bev. Also, NPs‐IgG was synthesized as a control probe.

### Nanoparticle Characterization

The molecular weight of synthetic probe precursors was determined by mass spectrometry (Bruker Daltonics, MA, USA). Particle size was determined by transmission electron microscopy (JEOL, Japan). Gel electrophoresis (BIO‐RAD, CA, USA), zeta potentiometer monitoring (Malvern, UK) the potential of the probe and multispectral laser imaging system (Azure Sapphire, USA) used to detect the conjugation of NPs and antibody was successful. Gd content in solution was determined by inductively coupled plasma optical emission spectrometry (Thermo Fisher Scientific, USA), and Gd content in mouse tissue was calculated. Absorption spectra were analyzed using a Multiskan Spectrum Microplate Spectrophotometer (Thermo Fisher Scientific, USA).

### Cellular Uptake

MDA‐MB‐231, T‐47D, or MCF10A cells were treated with NPs‐Bev or NPs‐IgG for different times and concentrations, stained with 4ʹ,6‐diamidino‐2‐phenylindole, and observed using fluorescence microscopy (Leica DM2700 P, USA).

### ROS Cell Levels

MDA‐MB‐231 cells were seeded in a 12‐well slide chamber at 1 × 10^5^ cells per well, cultured overnight, and incubated with/without NPs‐Bev ([Gd^3+^] = 25 × 10^−6^ m) for 4 h. Plates were then irradiated (or not) with X‐rays (6 Gy), and H2DCFDA concentrations which were used to assess ROS levels by using kits according to manufacturer's instructions (Thermo Fisher Scientific, USA). Fluorescence images were obtained using confocal microscopy (Zeiss, Germany) and analyzed using ImageJ software.

### Flow Cytometry

MDA‐MB‐231 cells were seeded in a 12‐well slide chamber at 1 × 10^5^ cells per well, cultured overnight, and incubated with/without NPs‐Bev ([Gd^3+^] = 25 × 10^−6^ m) for 4 h. Plates were then irradiated (or not) with X‐rays (6 Gy), and apoptosis quantified using an Annexin V binding kit (Beyotime, China) and flow cytometry. Flow cytometry was performed as previously described.^[^ ^42^ ^]^

### Ex Vivo and In Vivo MRI

Samples were separately prepared for the MRI phantom study. NPs‐Bev and NPs‐IgG were prepared at concentrations of 0.025, 0.05, 0.1, 0.2, and 0.4 mm with respect to Gd^3+^ ions in 1× PBS buffer. Deionized water was used as a control. Longitudinal relaxation times were measured to calculate sample relaxation rates. MDA‐MB‐231 cells bearing tumor mice were imaged by T1‐weighted MRI to evaluate NPs‐Bev as it specifically targeted tumors. Gd–DTPA and NPs‐IgG were used as controls. Animals were imaged using a 9.4T MRI scanner (Bruker, Germany). MRI signal intensities in regions of interest were tested upon the intravenous injection of NPs‐Bev, and at 0.5, 1, 2, 4, 12, 24 36, and 48 h postinjection. For quantitative comparisons, TBR ratios were calculated and analyzed using Radiant DICOM Veiver2020.2.

### NIR‐II Fluorescence Imaging and Biodistribution

Mice bearing subcutaneous MDA‐MB‐231‐Luc tumors (volume = 200–300 mm^3^) were randomly divided into two groups (*n* = 3) and injected with NPs‐Bev and NPs‐IgG through the tail vein at equivalent ICG doses (2.0 mg kg^−1^). Then, Bev blocking experiments were performed in vivo. MDA‐MB‐231‐Luc tumor‐bearing mice were randomized into two groups (*n* = 3) and injected with PBS or Bev (250 mg kg^−1^) at 30 min before NPs‐Bev injections (ICG = 2.0 mg kg^−1^). Mice were then anesthetized and fluorescence signals collected by the NIR‐II imaging system (Suzhou Yingrui Optical Technology Co., Ltd., China) at different times. NPs‐Bev ex vivo biodistribution was also calculated. MDA‐MB‐231‐Luc tumor‐bearing mice were intravenously injected with NPs‐Bev and humanely euthanized at 36 h to collect and visualize tumors and major organs for NIR‐II imaging analysis.

### Fluorescence‐Image‐Guided Surgery in a Multiple‐Microtumor Model

In mice bearing 30–60 mm^3^ MDA‐MB‐231‐Luc microtumors, bioluminescence imaging was used to calculate the number of microtumors. Then, mice were injected with NPs‐Bev through the tail vein (ICG = 2.0 mg kg^−1^) and NIR‐II fluorescence imaging performed at 36 h postinjection, and the number of microtumors calculated. Consistency of bioluminescence and fluorescence imaging in calculating tumor number and pathology was the gold standard.

### Fluorescence‐Image‐Guided Surgery in MDA‐MB‐231‐Luc Tumor‐Bearing Mice

Mice bearing 300–400 mm^3^ MDA‐MB‐231‐Luc tumors were randomized into two groups (*n* = 8); a visible light navigation (VL) surgical group and a NIR‐II navigation surgical group (NIR‐II). Groups were intravenously injected with NPs‐Bev (equivalent ICG dose = 2.0 mg kg^−1^). Tumors were resected in the NPs‐Bev group at 36 h postinjection. In the NIR‐II group, mice were placed under a NIR‐II imaging navigation surgical instrument for tumor resection. If residual fluorescence was detected, the tumor was removed until no residual fluorescence signals remained. In vivo mean fluorescence intensity (MFI) information was collected and analyzed. In the VL group, mouse tumors were surgically removed under visible light without surgical navigation. Mice were monitored every other day for tumor recurrence. Bioluminescence was performed at 35 days postinjection and mice with tumor recurrence volumes > 1500 mm^3^ and experiencing 25% body weight loss were humanely euthanized.

### Fluorescence Imaging in Spontaneous Breast Cancer Transgenic Mice

6–8 weeks old MMTV‐PyVT spontaneous breast cancer mice (*n* = 3) and wild‐type mice (*n* = 3) were intravenously injected with NPs‐Bev (ICG = 2.0 mg kg^−1^). Mice were humanely euthanized and NIR‐II fluorescence imaging performed at 36 h postinjection. Then, the 1st–5th breast tissue of both groups were resected, fluorescence imaging performed using NIR‐II imaging, and in vitro tumor MFI calculated. Histopathologies were assessed by two breast cancer specialists, and receiver operator characteristic (ROC) curves were fitted using MFI data and histopathology results. Additionally, 10 µm paraffin block slices were imaged using fluorescence flatbed scanning (Odyssey CLx, USA). Finally, adjacent 4 µm sections were prepared for H&E and IHC (VEGF‐A) staining to evaluate correlations between fluorescence intensity and histology.

### RT Sensitization in MDA‐MB‐231 Tumor Bearing Mice

MDA‐MB‐231 tumor bearing mice (50–100 mm^3^) were randomly divided into four groups (*n* = 5), intravenously injected with PBS and NPs‐Bev ([Gd^3+^] = 40 × 10^−6^ m kg^−1^), and irradiated (or not) with X‐rays on days 0 and 6. Irradiated mice received X‐ray RT (6 Gy × 2) at 12 h after injection, and tumor volumes were measured at consecutive 3 days intervals. At day 21, mice were humanely sacrificed and tumor volumes measured. Tumor tissues were excised, embedded in paraffin, sectioned into 4 µm slices for H&E, IHC stained with Ki‐67 (MAB‐0672, MXB Biotechnologies, China) and Caspase 3 (200270‐T08, SinoBiological, China), and images analyzed using ImageJ software.

### Western Blotting

Western blotting was performed as previously described.^[^ ^43^ ^]^ Primary antibodies were VEGF‐A (EP1176Y, Abcam, USA) and Tubulin (11224‐1AP, Proteintech, USA) (loading control). Secondary antibody was anti‐rabbit IgG (7074s, CST, USA).

### Cell Counting Kit‐8 Assay

MDA‐MB‐231 cells were seeded at 1 × 10^3^ cells per well in 96‐well microplates, cultured overnight, and incubated with/without NPs‐Bev ([Gd^3+^] = 25 × 10^−6^ m) for 4 h. Then, plates were irradiated (or not) with X‐rays (6 Gy). Cell counting kit‐8 (Beyotime, China) was performed as previously described.^[^ ^44^ ^]^ At least three separate experiments were conducted.

### Colony Formation Assay

MDA‐MB‐231 cells were seeded at 600 cells per well in 6‐well plates, cultured overnight, and then incubated with/without NPs‐Bev ([Gd^3+^] = 25 × 10^−6^ m) for 4 h. Then, plates were irradiated (or not) with X‐rays (6 Gy). After 2 weeks, cells were stained with Gentian Violet (Beyotime, China) and counted. Three independent experiments were performed.

### NPs‐Bev Biosafety

NPs‐Bev or PBS was injected into healthy BALB/C mice through the tail vein. Mice were reared in a normal barrier environment and body weights monitored. Peripheral blood was collected at days 1, 3, 7, and 28 after probe injection, and liver and kidney function indices examined. Mice were sacrificed by cervical dislocation, and the main organs removed for H&E staining to observe if histological morphology and pathological changes such as necrosis and degeneration had occurred. Liver and kidney functions and routine blood indices such as ALT, AST, CK‐MB, UREA, CREA, WBC, Lymph, Mon#, Gran#, RBC, HGB, and PLT were compared with control mice (injected with PBS).

### Statistical Analysis

SPSS 19.0 (SPSS Inc., Chicago, IL, USA) was used for statistical analysis. Differences between variables were measured using Student's *t*‐tests, Pearson's chi‐squared tests, and Spearman's log‐rank tests. Statistical significance was accepted at *p* < 0.05.

## Conflict of Interest

The authors declare no conflict of interest.

## Supporting information

Supporting Information

## Data Availability

The data that support the findings of this study are available from the corresponding author upon reasonable request.
