# Supplementary material for: Novel Self‐Assembled Multifunctional Nanoprobes for Second‐Near‐Infrared‐Fluorescence‐Image‐Guided Breast Cancer Surgery and Enhanced Radiotherapy Efficacy
Source: Adv Sci (Weinh). 2023 Jan 31;10(10):2205294. doi: 10.1002/advs.202205294 (PMC10074043; doi:10.1002/advs.202205294)
Supplement: Supplementary file 1 — Supporting Information [file ADVS-10-2205294-s001.pdf]

ADVANCED SCIENCE
Open Access

Supporting Information

for *Adv. Sci.*, DOI 10.1002/advs.202205294

Novel Self-Assembled Multifunctional Nanoprobes for Second-Near-Infrared-Fluorescence-Image-Guided Breast Cancer Surgery and Enhanced Radiotherapy Efficacy

*Yong-Qu Zhang, Wan-Ling Liu, Xiang-Jie Luo, Jun-Peng Shi, Yun-Zhu Zeng, Wei-Ling Chen, Wen-He Huang, Yuan-Yuan Zhu, Wen-Liang Gao, Rong-Hui Li, Zi-He Ming, Li-Xin Zhang, Rui-Qin Yang, Jia-Zheng Wang and Guo-Jun Zhang**

Supporting Information

Novel self-assembled multifunctional nanoprobes for second near-infrared-fluorescence image-guided breast cancer surgery and enhanced radiotherapy efficacy

This file includes:

Supplementary Figures S1–S16

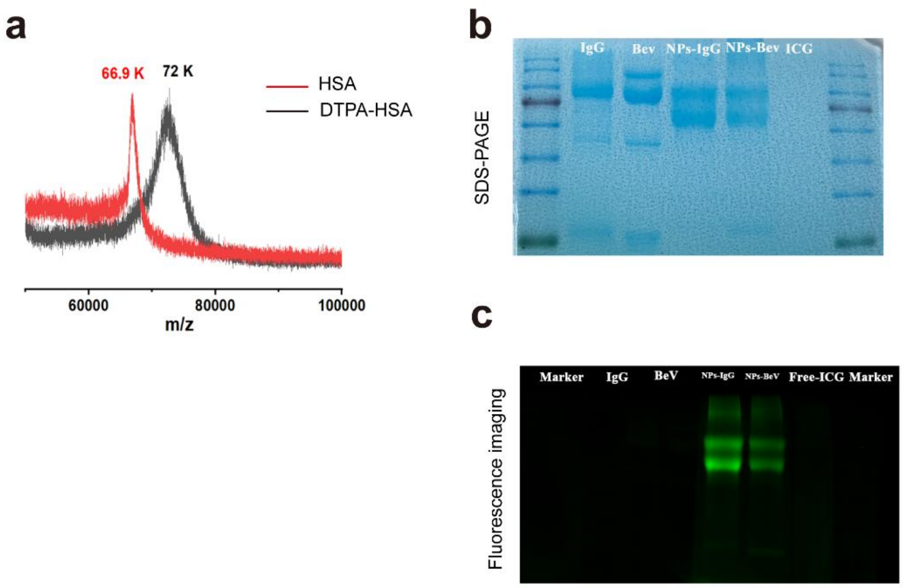

Figure S1. Characterization of the study probe and its components

(**a**) DTPA-HSA conjugates were characterized by MALDI-TOF mass spectrometry. An increase in molecular weight was observed from 66.9 kDa (native HSA, red line) to 72 kDa (DTPA-HAS, black line). **(b, c)** Sodium dodecyl sulfate-polyacrylamide gel electrophoresis (**b**) and multispectral laser fluorescence imaging (**c**) show the conjugation between NPs and the Bevacizumab antibody was successful.

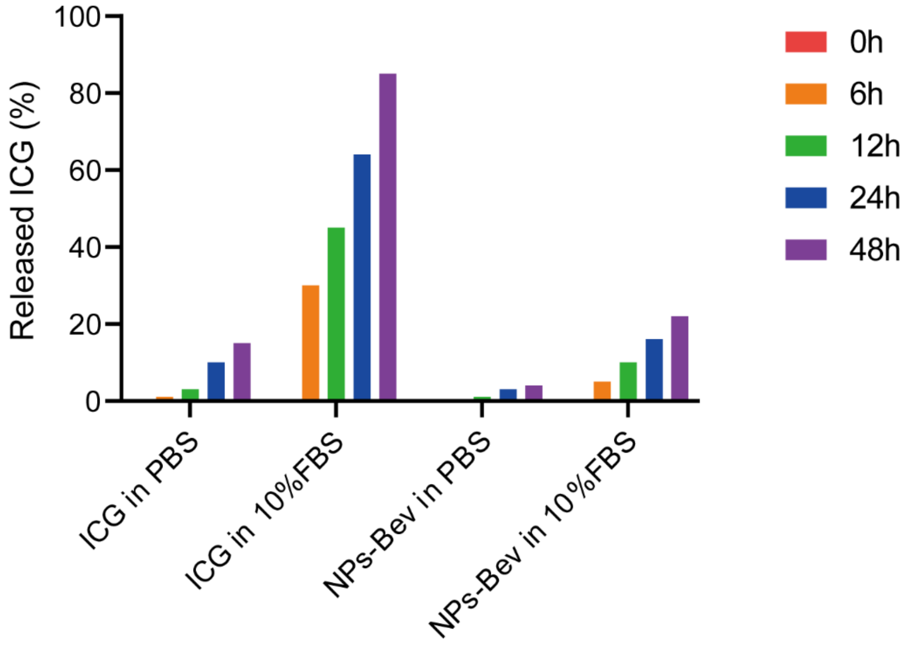

Figure S2. Stability profile of ICG in NPs-Bev

We added 1 ml NPs-Bev solution (ICG = 0.5 mg) to a dialysis bag (molecular weight cut-off = 10 kDa) and dialyzed it against 20 ml PBS or PBS plus 10% fetal bovine serum (FBS). Also, 1 ml ICG aqueous solution (ICG = 0.5 mg) was used as a control. For NPs-Bev and free ICG, ICG release was low due to ICG aggregation in PBS. However, in 10% FBS, ICG release from NPs-Bev solution was much lower when compared with free ICG at different times, indicating that ICG was stably embedded in NPs-Bev, with minimal leakage into the physiological environment.

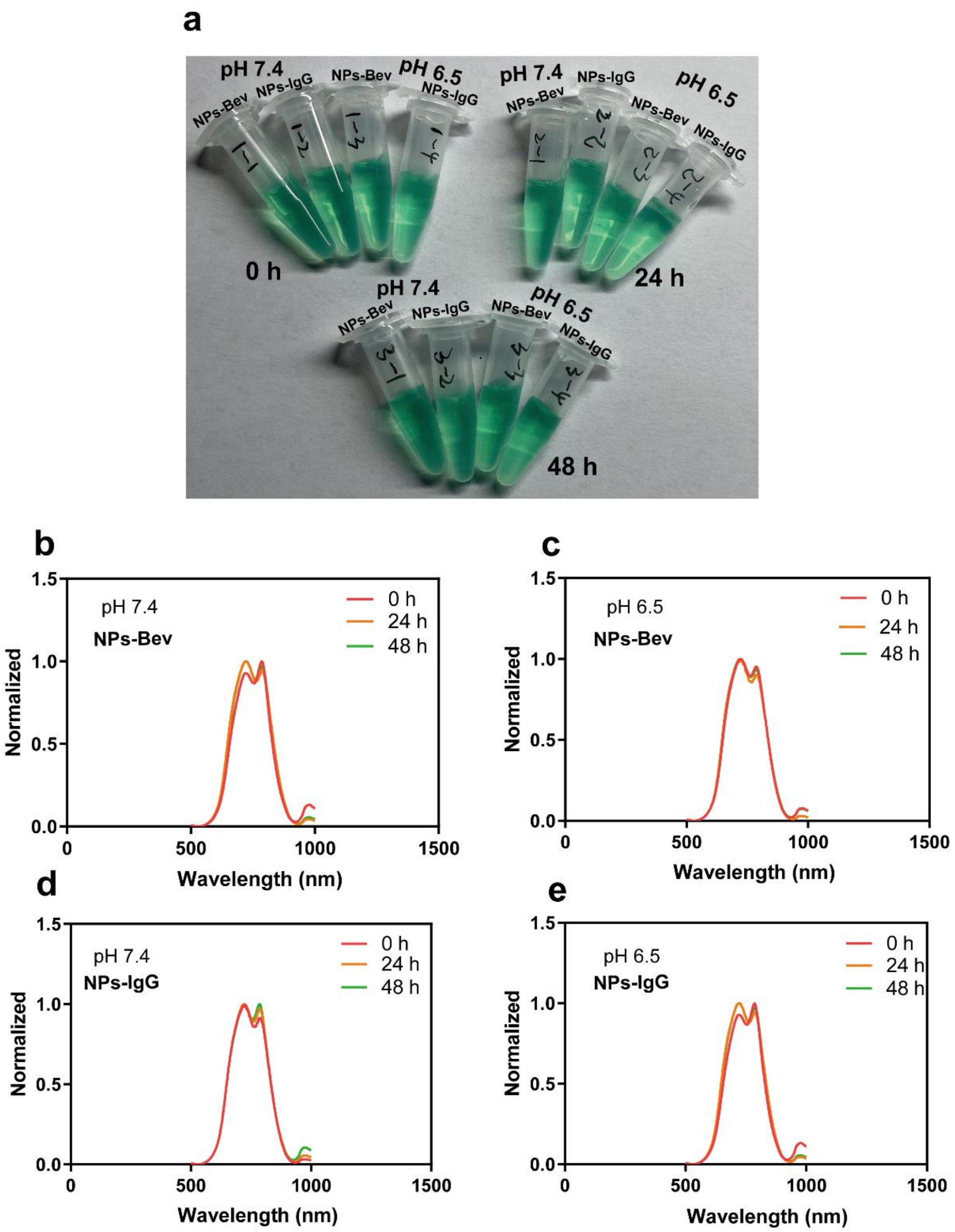

Figure S3. Fluorescence absorption analyses showing NPs-Bev and NPs-IgG stability in PBS at different pHs.

(**a**) NPs-Bev and NPs-IgG were dissolved in PBS at pH 7.4 and pH 6.5 under visible light, (**b, c**) The fluorescence absorption stability of NPs-Bev was examined in PBS at pH 7.4 (**b**) or pH 6.5 (**c**). (**d, e**) Fluorescence absorption stability of NPs-IgG was examined in PBS at pH 7.4 (**d**) or pH 6.5 (**e**).

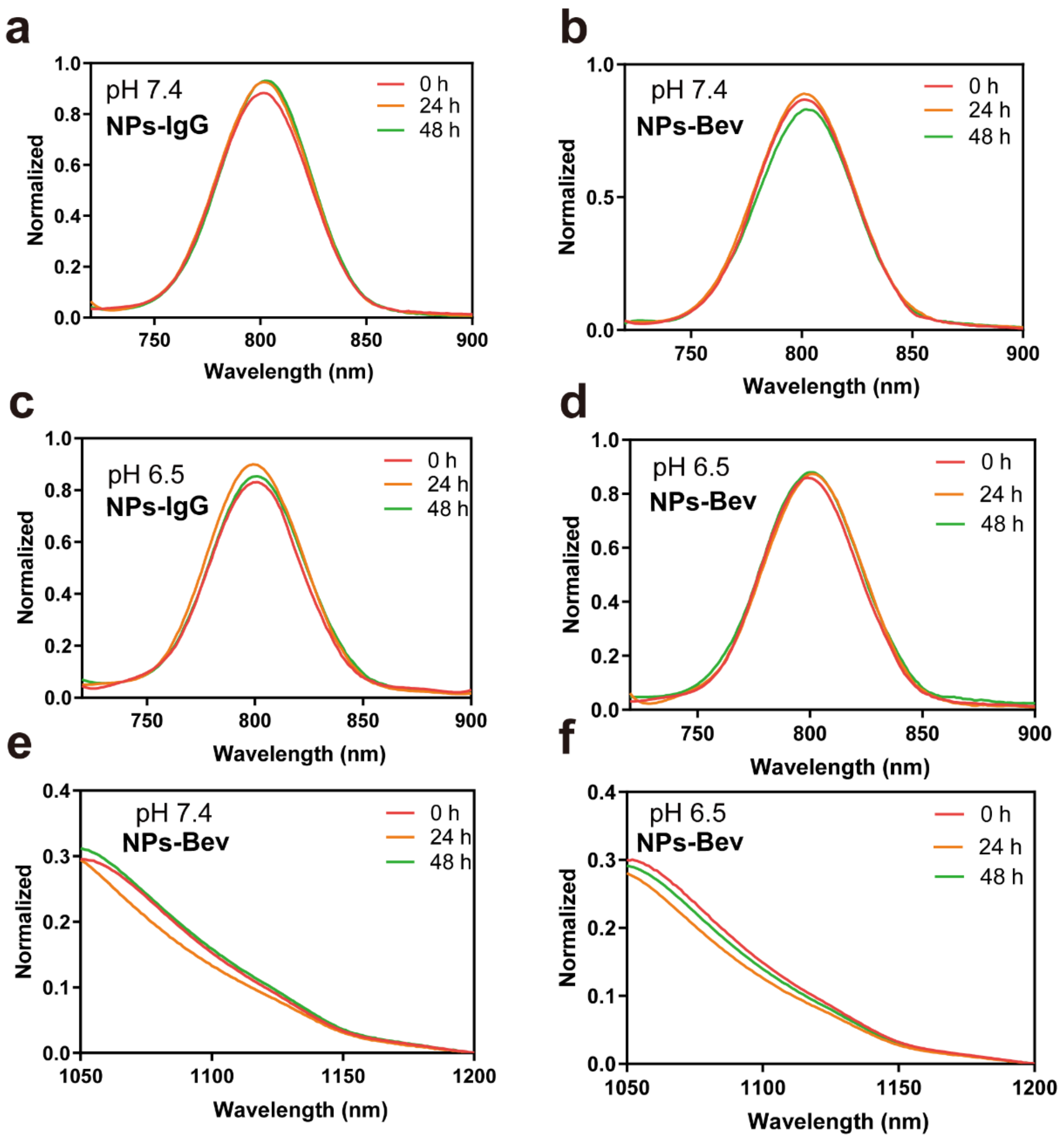

Figure S4. Fluorescence emission analyses of NPs-Bev and NPs-IgG stability in PBS at different pHs.

(**a, b**) Fluorescence emission stability of NPs-IgG (**a**) and NPs-Bev (**b**) in PBS at pH 7.4. (**c, d**) Fluorescence emission stability of NPs-IgG (**c**) and NPs-Bev (**d**) in PBS at pH 6.5 (**e, f**) NIR-II fluorescence long trailing signals of NPs-Bev in PBS at pH 7.4 (**e**) or pH 6.5 (**f**).

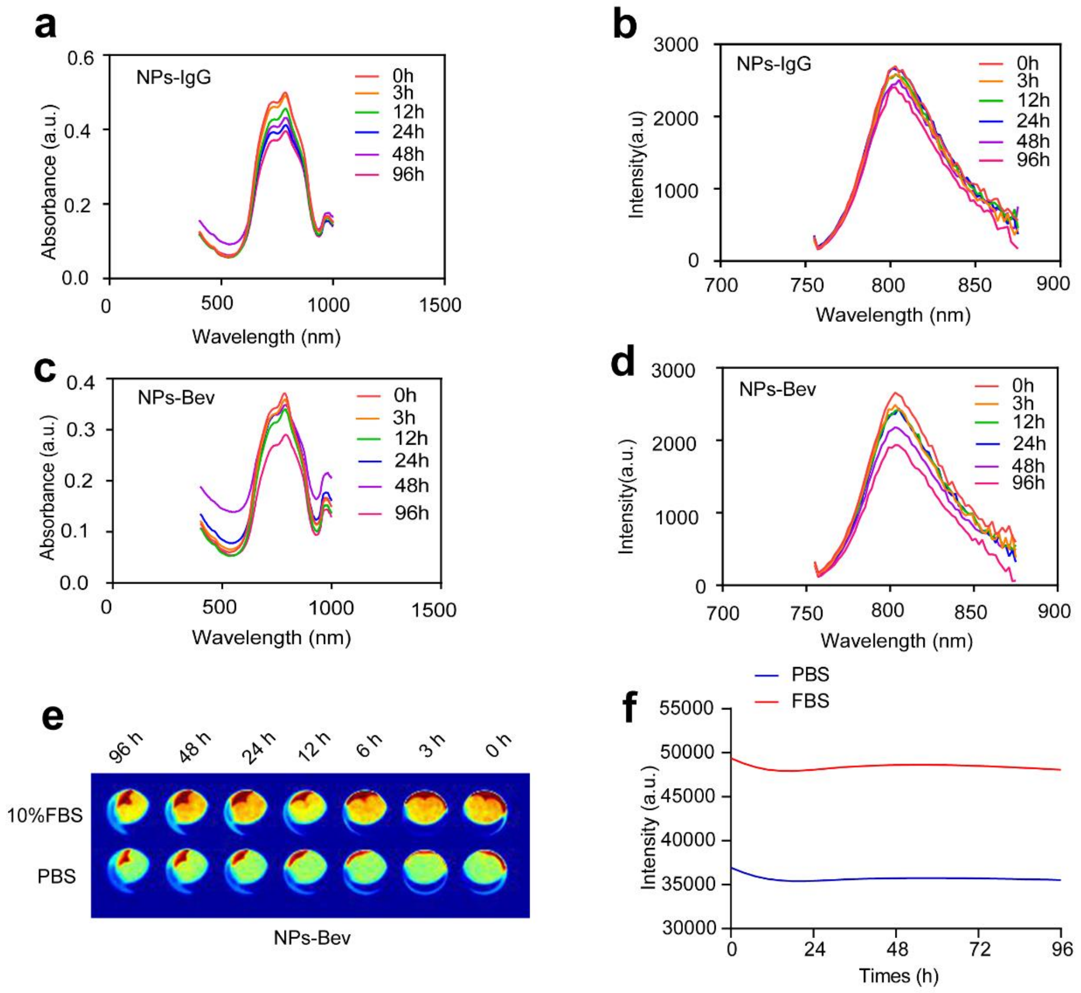

Figure S5. Characterization of NPs-Bev and NPs-IgG

(**a**, **c**) Fluorescence absorbance stability of NPs-IgG (**a**) and NPs-Bev (**c**) at different times (0, 3, 12, 24, 48, and 96 h) (**b, d**) Fluorescence intensity stability of NPs-IgG (**b**) and NPs-Bev (**d**) at different times (0, 3, 12, 24, 48, and 96 h) (**e, f**) Fluorescence intensity stability of NPs-Bev in PBS or 10% FBS for 96 h.

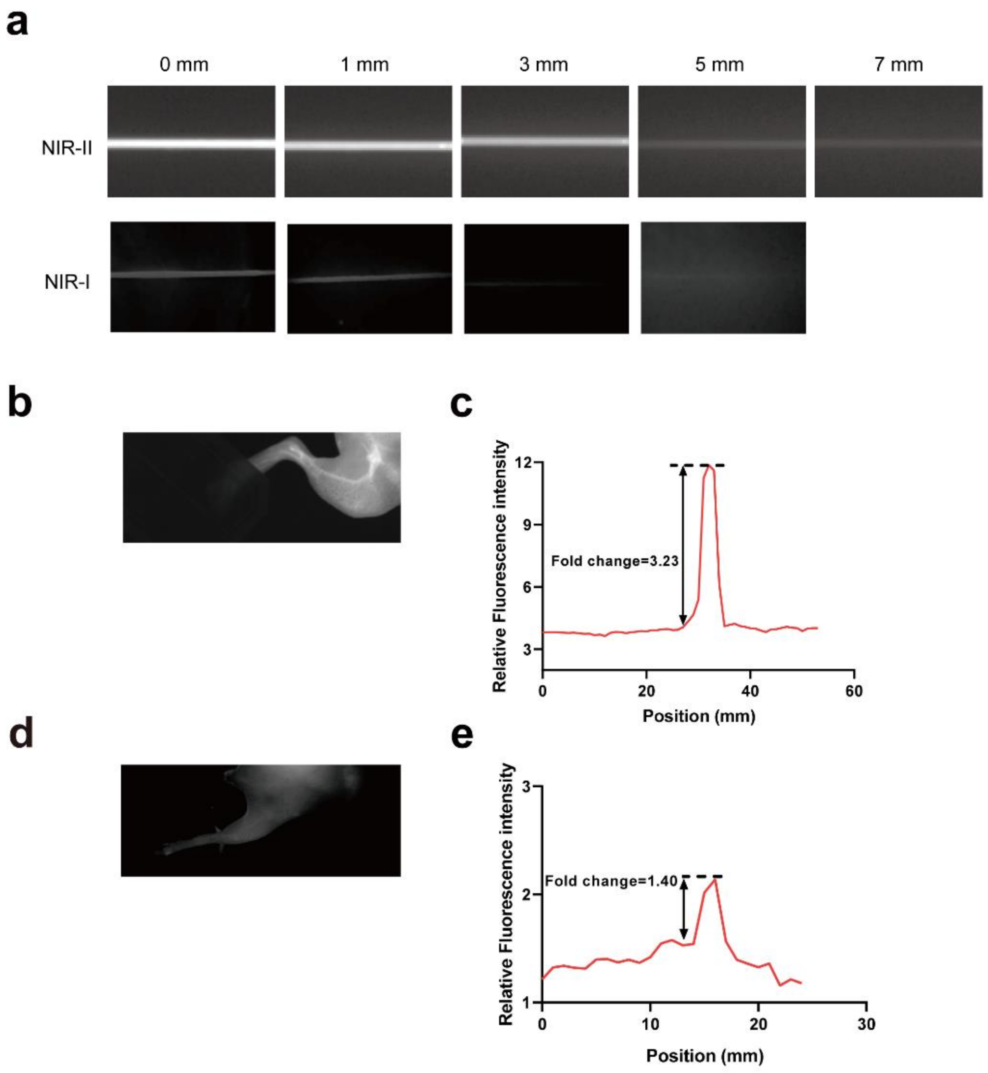

Figure S6. Penetration depth of ICG in NIR-I and NIR-II

(**a**) ICG penetrated up to 7 mm at NIR-II in 1% intralipid solution and only 5 mm at NIR-I in 1% intralipid solution (**b, d**) Fast whole body vascular imaging of BALB/C nude mice after injection with 100 µl ICG (1000 µg ml^{-1}) in NIR-II or NIR-I (**c, e**) Cross-sectional fluorescence intensity profile of tumor vessels in b and d with relative fluorescence fold changes of 3.23 (c) and 1.40 (**e**), respectively.

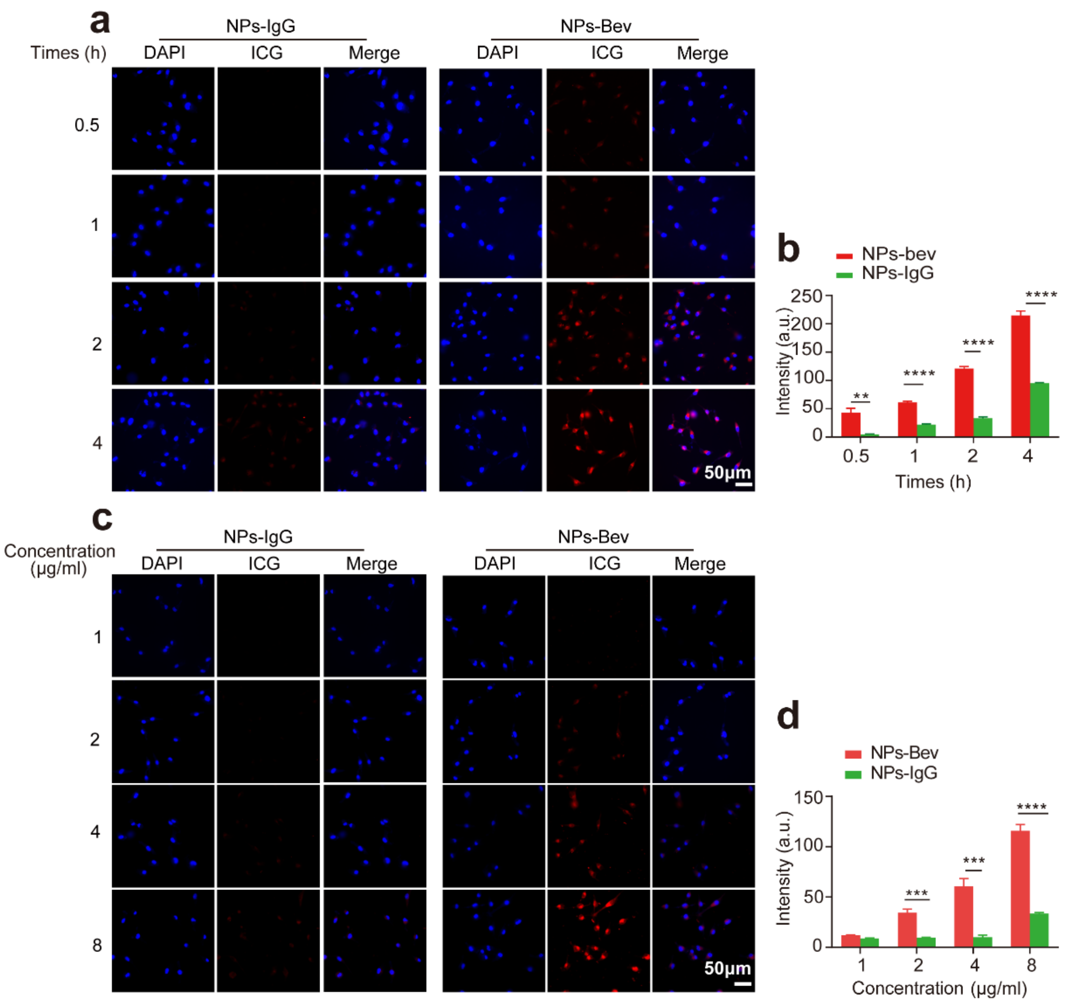

Figure S7. Optimal cell fluorescence uptake of NPs-IgG and NPs-Bev

Confocal laser scanning images of MDA-MB-231 cells incubated with NPs-IgG and NPs-Bev at different times (0.5, 1, 2, and 4 h) (**a, b**) and concentrations (1, 2, 4, and 8 μg/ml) (**c, d**) ***P*<0.01, ****P*<0.001, *****P*<0.0001. Scale bar = 50 μm.

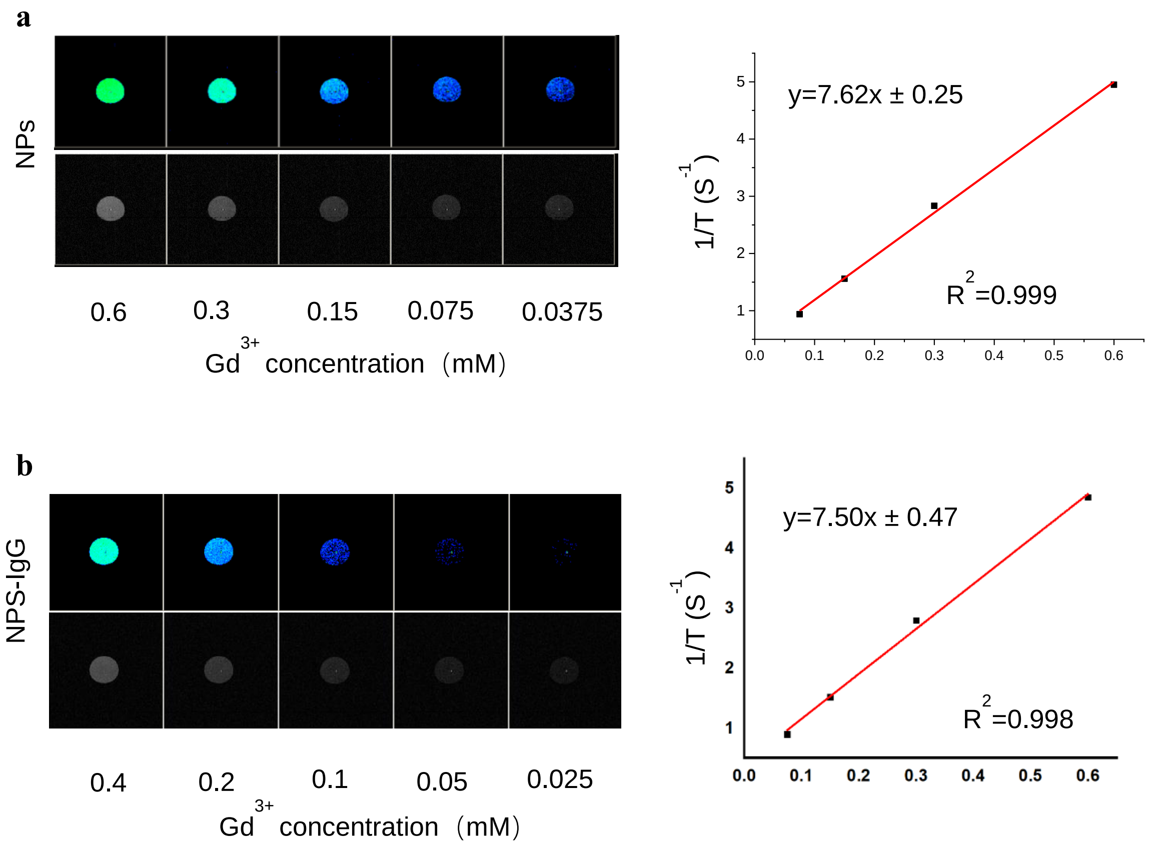

Figure S8. ***Ex vivo*** **MRI of NPs and NPs-IgG**

(**a**) T_1-weighted MR phantom imaging of ^{Gd}DTPA-HAS (NPs) (left) and longitudinal relaxivities (r1) for NPs recorded using a 0.5-T MRI scanner (right) (**b**) T1-weighted MR phantom imaging of NPs-IgG (left) and longitudinal relaxivities (r1) for NPs-IgG recorded using a 0.5-T MRI scanner (right).

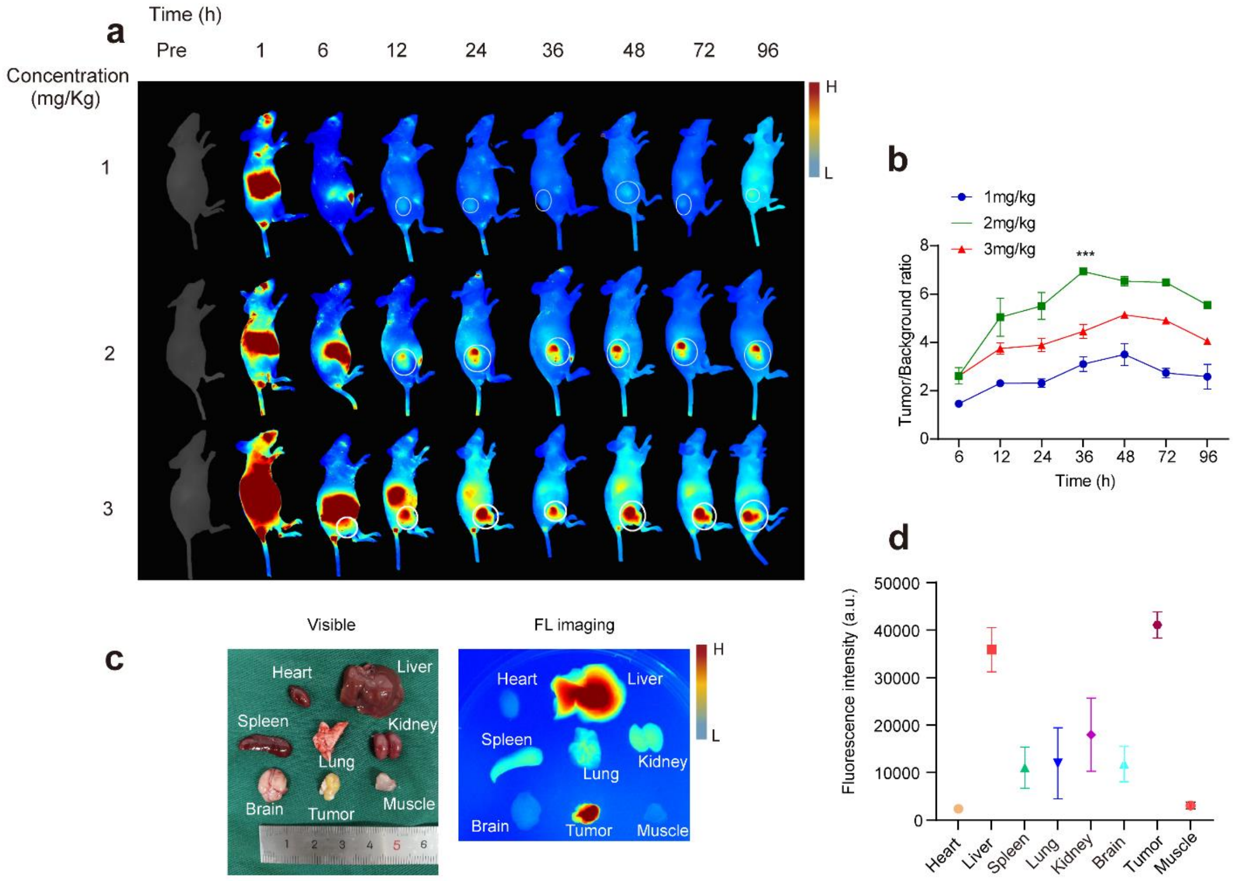

Figure S9. Optimal *in vivo* imaging showing concentration and biological distribution of NPs-Bev

(**a**) Optimal *in vivo* imaging showing different concentrations of NPs-Bev. (**b**) The 2 mg kg^{-1} concentration at 36 h post-injection had the highest tumor/background ratio (TBR). (**c**) The biological distribution of NPs-Bev in MDA-MB-231 tumor-bearing mice at 36 h post-injection (Left: Visible, Right: fluorescence (FL) imaging). (**d**) Statistical analysis showing fluorescence signals were strongest in the tumor, followed by the liver.

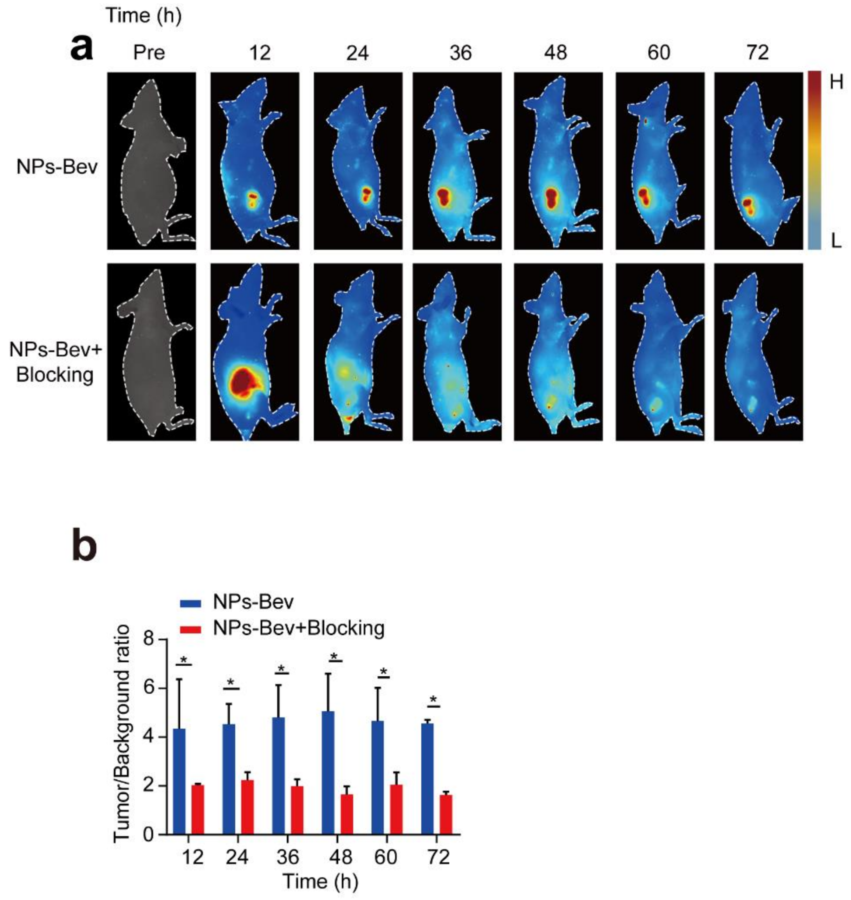

Figure S10. ***In vivo*** **bevacizumab blocking experiments**

(a) MDA-MB-231-Luc tumor-bearing mice were randomly divided into two groups (n=3) and intravenously injected with PBS or bevacizumab block (250 mg kg^{-1}) at 30 min before NPs-Bev injection at the equivalent ICG dose (2.0 mg kg^{-1}). (b) TBR analysis in groups with or without bevacizumab block. *P<0.05

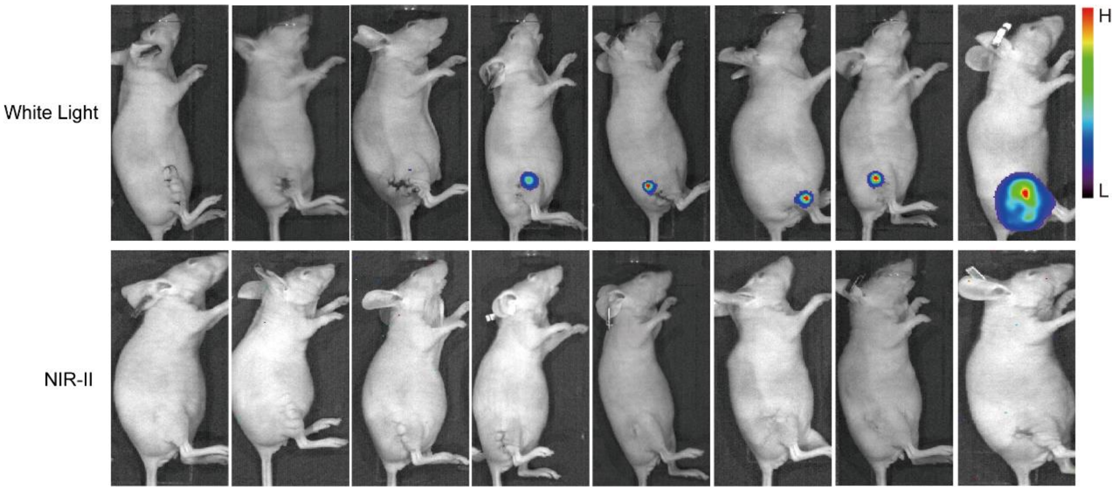

Figure S11. Postoperative follow-up of NPs-Bev guided by NIR-II

Upper panel: Bioluminescence after 35 days in the White Light operation group

Lower panel: Bioluminescence after 35 days in the NPs-Bev operation group

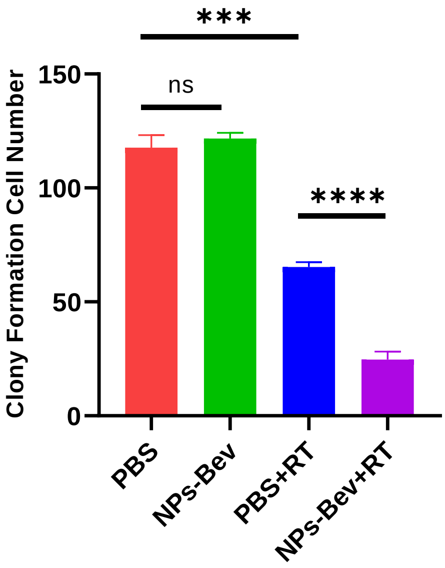

Figure S12. Quantitative analysis of MDA-MB-231 colony formation assays after irradiation (6 Gy) with PBS and NPs-Bev, with or without X-rays. ****P*<0.01, *****P*<0.0001, ns: Not Statistically Significant.

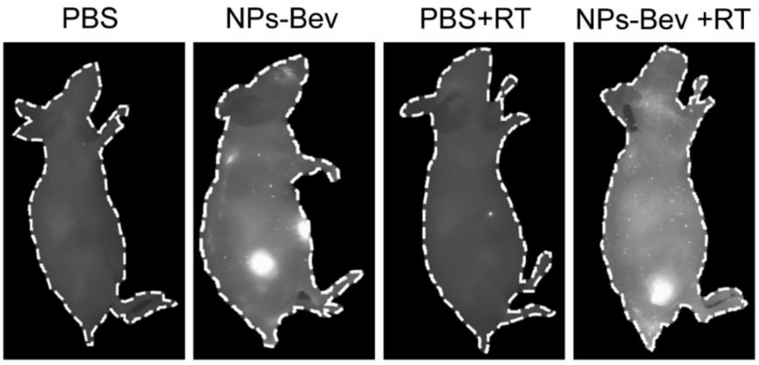

Figure S13. Representative images showing fluorescence in mice before treatment with NPs-Bev and PBS, with or without X-ray radiation therapy (RT). PBS: PBS without X-ray RT, NPs-Bev: NPs-Bev without X-ray RT, PBS+RT: PBS with X-ray RT, NPs-Bev+RT: NPs-Bev with X-ray RT. RT was conducted on days 0 and 6, and each exposure was 6 Gy.

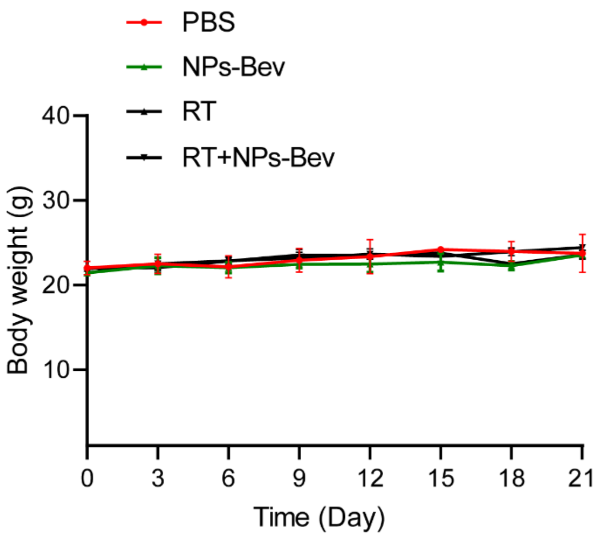

Figure S14. Body weight curves for mice treated with NPs-Bev and PBS, with or without X-ray RT (n=5).

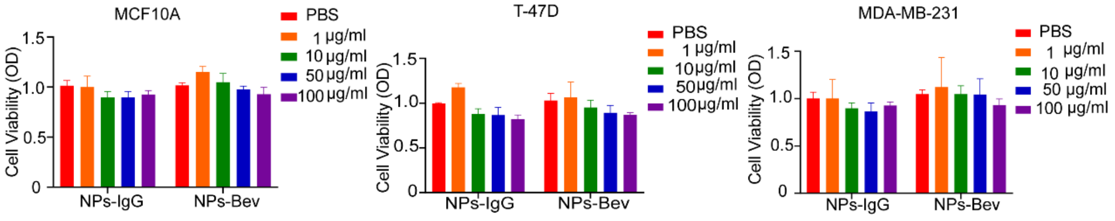

Figure S15. ***Ex vivo*** **cytotoxicity of NPs-IgG and NPs-Bev.** Normal mammary epithelial cell MCF-10A viability (**a**), T-47D viability (**b**), and MDA-MB-231 cell

viability (**c**) after incubation with NPs-IgG and NPs-Bev gradient concentrations using the CCK-8 assay.

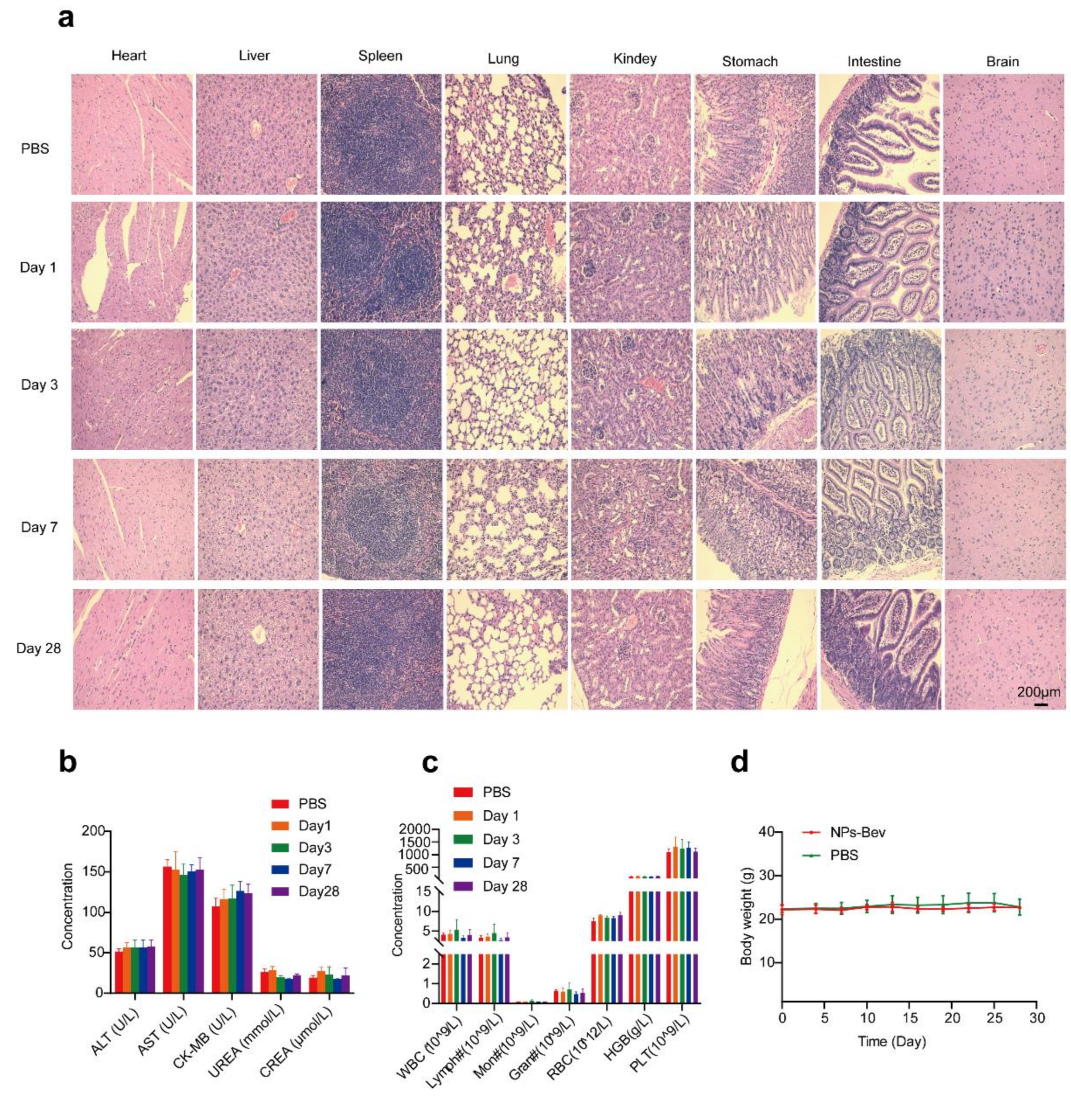

Figure S16. **NPs-Bev biosafety**

(**a**) H&E staining of vital organs (heart, liver, spleen, lung, kidney, and brain) from BALB/c mice at different times (1, 3, 7, and, 28 days) after intravenous injection with NPs-Bev or PBS. (**b**) Serum biochemistry results (**c**) and blood cytology at different times (days 1, 3, 7, and, 28) after intravenous injection with NPs-Bev or PBS (**d**) Body weight curves of mice treated intravenously with PBS or NPs-Bev (n=3). alanine aminotransferase (ALT), aspartate aminotransferase (AST), creatine kinase MB

(CK-MB), UREA, and creatinine (CREA), white blood cell (WBC), lymphocyte (Lymph), monocyte cell(Mon#), neutrophil granulocyte (Gran#), red blood cell (RBC), hemoglobin (HGB) and platelets (PLT). Scale bar = 200 μm.
